# Molecular Epidemiology of Colonizing and Infecting Isolates of *Klebsiella pneumoniae*

**DOI:** 10.1128/mSphere.00261-16

**Published:** 2016-10-19

**Authors:** Rebekah M. Martin, Jie Cao, Sylvain Brisse, Virginie Passet, Weisheng Wu, Lili Zhao, Preeti N. Malani, Krishna Rao, Michael A. Bachman

**Affiliations:** a Department of Pathology, University of Michigan Medical School, Ann Arbor, Michigan, USA; b Institut Pasteur, Microbial Evolutionary Genomics, Paris, France; c BRCF Bioinformatics Core, University of Michigan, Ann Arbor, Michigan, USA; d Department of Biostatistics, School of Public Health, University of Michigan, Ann Arbor, Michigan, USA; e Division of Infectious Diseases, Department of Internal Medicine, University of Michigan Medical School, Ann Arbor, Michigan, USA; f Section of Infectious Diseases, Veterans Affairs Ann Arbor Healthcare System, Ann Arbor, Michigan, USA; JMI Laboratories

**Keywords:** Klebsiella, MLST, cgMLST, colonization, infection, pneumonia, whole-genome sequencing, wzi

## Abstract

*Klebsiella pneumoniae* is among the most common causes of hospital-acquired infections and has emerged as an urgent threat to public health due to carbapenem antimicrobial resistance. *K. pneumoniae* commonly colonizes hospitalized patients and causes extraintestinal infections such as urinary tract infection, bloodstream infection (septicemia), and pneumonia. If colonization is an intermediate step before infection, then detection and characterization of colonizing isolates could enable strategies to prevent or empirically treat *K. pneumoniae* infections in hospitalized patients. However, the strength of the association between colonization and infection is unclear. To test the hypothesis that hospitalized patients become infected with their colonizing strain, 1,765 patients were screened for rectal colonization with *K. pneumoniae*, and extraintestinal isolates from these same patients were collected over a 3-month period in a cohort study design. The overall colonization prevalence was 23.0%. After adjustment for other patient factors, colonization was significantly associated with subsequent infection: 21 of 406 (5.2%) colonized patients later had extraintestinal infection, compared to 18 of 1,359 (1.3%) noncolonized patients (adjusted odds ratio [OR], 4.01; 95% confidence interval, 2.08 to 7.73; *P* < 0.001). Despite a high diversity of colonizing isolates, 7/7 respiratory, 4/4 urinary, and 2/5 bloodstream isolates from colonized patients matched the patient corresponding rectal swab isolates, based on *wzi* capsular typing, multilocus sequence typing (MLST), and whole-genome sequence analysis. These results suggest that *K. pneumoniae* colonization is directly associated with progression to extraintestinal infection.

**IMPORTANCE** K. pneumoniae commonly infects hospitalized patients, and these infections are increasingly resistant to carbapenems, the antibiotics of last resort for life-threatening bacterial infections. To prevent and treat these infections, we must better understand how *K. pneumoniae* causes disease and discover new ways to predict and detect infections. This study demonstrates that colonization with *K. pneumoniae* in the intestinal tract is strongly linked to subsequent infection. This finding helps to identify a potential time frame and possible approach for intervention: the colonizing strain from a patient could be isolated as part of a risk assessment, and antibiotic susceptibility testing could guide empirical therapy if the patient becomes acutely ill.

## Abstract (precis)

*K. pneumoniae* commonly infects hospitalized patients, and these infections are increasingly resistant to carbapenems, the antibiotics of last resort for life-threatening bacterial infections. To prevent and treat these infections, we must better understand how *K. pneumoniae* causes disease and discover new ways to predict and detect infections. This study demonstrates that colonization with *K. pneumoniae* in the intestinal tract is strongly linked to subsequent infection. This finding helps to identify a potential time frame and possible approach for intervention: the colonizing strain from a patient could be isolated as part of a risk assessment, and antibiotic susceptibility testing could guide empirical therapy if the patient becomes acutely ill.

## INTRODUCTION

*Klebsiella pneumoniae* is a Gram-negative bacillus and a member of the *Enterobacteriaceae* family. *Klebsiella* spp. are among the most common causes of hospital-acquired infections (HAIs) in the United States and are responsible for about 10% of all infections (1). *K. pneumoniae* commonly infects the urinary tract, respiratory tract, surgical sites, and the bloodstream and can cause severe diseases such as pneumonia and sepsis (2). Complicating treatment of *K. pneumoniae* infections is the recent emergence of strains encoding extended-spectrum β-lactamases (ESBLs) (3) and *K. pneumoniae* carbapenemases (KPCs) (4). Because of their high associated mortality and potential for rapid spread, the Centers for Disease Control and Prevention (CDC) has declared carbapenem-resistant *Enterobacteriaceae* (CRE) an urgent threat to public health (5). A 2012-2013 epidemiological study found the annual incidence of CRE in seven U.S. states was 2.93 per 100,000 people, with most cases found in individuals with underlying comorbidities or previous exposure to health care (6).

The major sources of *K. pneumoniae* that cause HAIs remain unclear. Intestinal colonization (7), the presence of *K. pneumoniae* in the environment, contaminated instruments (8), and healthcare workers’ hands (8, 9) have all been implicated in transmission. *K. pneumoniae* gastrointestinal colonization rates in hospitalized patients are estimated to be 20 to 38%, based largely on studies conducted before 1980 (2, 7, 10, 11), and a more recent study identified a 21.1% fecal carriage rate in healthy adults in Korea (12) with a high proportion of sequence type 23 (ST23) isolates that were associated with pyogenic liver abscess. Prior treatment with antimicrobials has been reported as a risk factor for colonization (7, 13, 14), but this factor may be specific for antimicrobial-resistant *Klebsiella*. Earlier work identified gastrointestinal colonization with *K. pneumoniae* as a reservoir for infection with *K. pneumoniae* (7), but such colonization may reflect the virulence potential of two predominant serotypes in the cohort. Regardless of transmission route, *K. pneumoniae* appears to be transmitted efficiently, as evidenced by reported outbreaks (15).

New techniques in molecular strain typing offer the opportunity to measure concordance among colonizing and infecting isolates of *K. pneumoniae* in patients. Repetitive sequence-based PCR (rep-PCR) has been widely used to characterize isolates of antibiotic-resistant *K. pneumoniae* (16–18). Multilocus sequence typing (MLST) has been used to characterize *K. pneumoniae* based on polymorphisms of seven conserved genes (*rpoB*, *gapA*, *mdh*, *pgi*, *phoE*, *infB*, and *tonB*) (19), and it is widely used as a common language for *K. pneumoniae* strain typing. Sequencing of the *wzi* gene is a rapid and inexpensive approach to differentiate *K. pneumoniae* capsular types (20). Recent studies reported that *wzi* sequencing has a similar discriminatory power to MLST (21–23), suggesting that *wzi* could be used as a rapid and inexpensive method to screen for genetic differences among strains. As probably the most discriminatory method, whole-genome sequencing (WGS) is even able to distinguish isolates from the same lineage evolving in a single patient (15). For *K. pneumoniae*, a core genome MLST scheme, based on WGS and 634 conserved genes, has been validated as a way to characterize strains in a systematic and reproducible manner. These new tools provide methods to both screen for strain differences and confirm strain concordance with the power of WGS (24).

The objective of this study was to test the hypothesis that intestinal colonization leads to subsequent infection with *K. pneumoniae* in hospitalized patients. To test this hypothesis, we determined the association and strain concordance between intestinal *K. pneumoniae* colonization and subsequent extraintestinal infections in a large cohort. The rationale for this study was that, if colonizing isolates are highly likely to cause disease, they can provide a focus for pathogenesis research and a potential window for infection prevention interventions.

## RESULTS

### Patient demographics.

**FIG 1:**
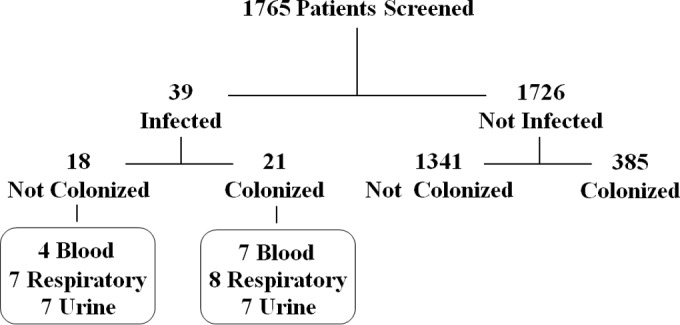
Study population. Adult patients in the University of Michigan Health System’s ICU and adult hematology/oncology patients were screened for colonization and extraintestinal infection with *K. pneumoniae* between July and October 2014 (*n* = 1,765), divided into “infected” and “not infected” groups, and further divided into “colonized” and “not colonized.” The number of infections by body site are shown in boxes; one colonized patient met case definitions for both pneumonia and UTI.

During a 3-month period, 1,800 patients were screened for *K. pneumoniae* colonization by rectal swab culture; extraintestinal infection with *K. pneumoniae* among this group was assessed based on positive clinical cultures. After excluding 35 patients whose first rectal swabs were collected after their first positive *K. pneumoniae* isolate at an extraintestinal site, a total of 1,765 patients were included in subsequent analysis (Fig. 1). Of 77 patients with a positive blood, respiratory, or urine culture, 39 patients met case definitions of infection (11 cases of bloodstream infection [BSI], 15 pneumonia cases, and 14 with urinary tract infection [UTI]; 1 patient met case definitions for both pneumonia and a UTI). The demographic characteristics of patients with and without clinical infections are shown in Table 1. There were no significant differences in age, sex, or self-reported race/Hispanic ethnicity. Antibiotic exposure was numerically higher in the uninfected group (26.2% versus 12.8% of infected patients), but the difference did not reach statistical significance (*P* = 0.067). Neurologic disorders and fluid and electrolyte disorders were significantly more frequent in infected patients than in noninfected patients. Baseline albumin levels were significantly lower in the infected group (*P* = 0.009), and length of stay was significantly longer in the infected group (14.9 versus 11.6 days for uninfected; *P* = 0.01).

**TABLE 1:** Demographic characteristics of patients with and without infection

Variable	No. (%) or mean ± SD		*P* value^a^
	Infected (*n* = 39)	No infection (*n* = 1,726)	
Female	19 (48.7)	835 (48.3)	>0.99
White race	31 (79.5)	1,438 (83.3)	0.488
Hispanic	0 (0)	33 (1.9)	0.984
Prior admit (28 days)	27 (69.2)	947 (54.8)	0.079
Length of stay (days)	14.9 ± 14.9	11.6 ± 18.9	0.01
Neurologic disorder^b^	6 (15.4)	109 (6.3)	0.029
Fluid and electrolyte disorder^b^	20 (51.3)	569 (33)	0.019
Renal disease^b^	5 (12.8)	286 (16.6)	0.533
Liver disease^b^	5 (12.8)	112 (6.5)	0.123
Alcohol abuse^b^	1 (2.6)	94 (5.4)	0.442
Solid organ tumor^b^	13 (33.3)	403 (23.3)	0.151
Diabetes mellitus, uncomplicated^b^	8 (20.5)	242 (14)	0.218
*K. pneumoniae* colonization	21 (53.8)	385 (22.3)	<0.001
Central line before colonization	27 (69.2)	963 (55.8)	0.099
Antibiotic exposure^c^	5 (12.8)	453 (26.2)	0.067
Aminoglycoside	0 (0)	178 (10.3)	0.984
Fluoroquinolone	0 (0)	0 (0)	NA
Macrolide	2 (5.1)	151 (8.7)	0.433
Cephalosporin	1 (2.6)	139 (8.1)	0.237
Carbapenem	0 (0)	0 (0)	NA
Clindamycin	2 (5.1)	97 (5.6)	0.895
Age (yrs)	62.7 ± 12.8	58.2 ± 16.1	0.078
Hemoglobin, baseline (g/dl)	10.4 ± 2.4	11.1 ± 2.5	0.062
Platelets, baseline (×10^3^/µl)	167.9 ± 90.9	207.3 ± 115.5	0.012
Albumin, baseline (g/dl)	3.3 ± 0.6	3.5 ± 0.6	0.009
Body mass index (kg/m^2^)	27 ± 6.7	29.5 ± 9.5	0.128

a *P* values were obtained using Student’s *t* test for continuous variables and the chi-square or Fisher's exact test for categorical variables.

b As defined by the Elixhauser index (35).

c All classes combined, with receipt before colonization; selected individual classes are also listed.

### Association of colonization with *K. pneumoniae* and infection.

Of the 1,765 patients analyzed, 406 (23%) were identified as colonized (Table 2). Of those colonized, 5.2% (*n* = 21) later developed infection with *K. pneumoniae* at an extraintestinal site, compared to only 1.3% (*n* = 18) of noncolonized patients (unadjusted odds ratio [OR], 4.06; 95% confidence interval [CI], 2.14 to 7.7; *P* < 0.0001). In terms of specific sites, colonization was significantly associated with BSI (OR, 5.94; 95% CI, 1.73 to 20.41; *P* = 0.005), pneumonia (OR, 3.88; 95% CI, 1.40 to 10.77; *P* =. 01), and UTI (OR, 3.39; 95% CI, 1.18 to 9.72; *P* = 0.024) (Table 3). For 20 of 21 colonized patients who became infected, colonization was detected on their initial rectal swab; 1 patient became positive on their second rectal swab 6 days later.

**TABLE 2:** Prior colonization with *K. pneumoniae* versus subsequent infection

Infection status	No. (%) colonized	No. (%) not colonized	Total	OR (95% CI) for infection	*P* value^a^
Infection	21 (5.2)	18 (1.3)	39	4.06 (2.14–7.7)	<0.001
No infection	385 (94.8)	1,341 (98.7)	1,726		
Total	406 (23)	1,359 (77)	1,765		

a Determined using the Fisher's exact test.

**TABLE 3:** Association with prior colonization for each site of infection

Site of infection	Colonization frequency (%)		OR (95% CI)	*P* value^a^
	Infected	Not infected		
Blood	7/11 (64)	399/1,754 (23)	5.94 (1.73–20.41)	0.005
Respiratory	8/15 (53)	398/1,750 (23)	3.88 (1.40–10.77)	0.01
Urine	7/14 (50)	399/1,751 (23)	3.39 (1.18–9.72)	0.024

a *P* values were obtained using Fisher’s exact test.

**FIG 2:**
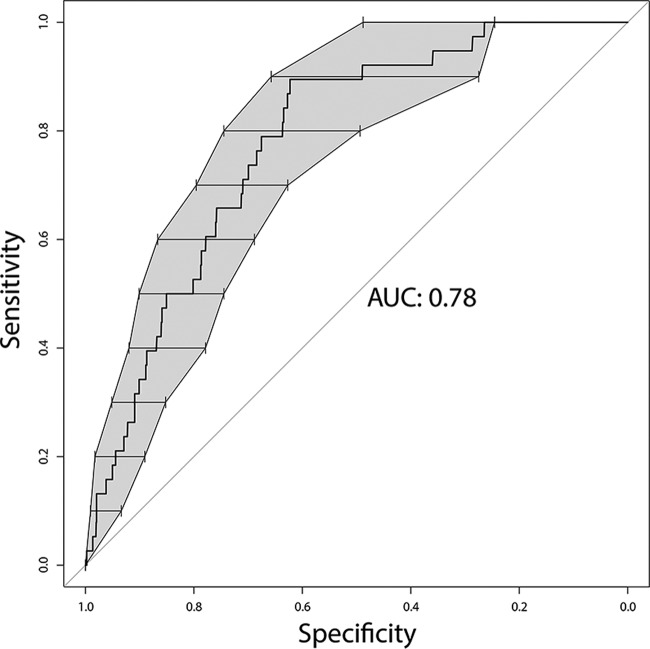
Receiver operator characteristic curve for a multivariable model of risk factors for clinical infection. Multiple logistic regression of *K. pneumoniae* infection was used to generate a predictive model using five patient variables (Table 4). Bars and shaded areas of ROC curves represent bootstrapped 95% confidence intervals (10,000 replicates) for specificity at each level of sensitivity (AUC, 0.78; 95% CI, 0.72 to 0.84).

In the final multivariable model, colonization with *K. pneumoniae* had the highest association with infection (all sites) after adjustment for other potential confounders (OR, 4.01; 95% CI, 2.08 to 7.73; *P* < 0.001) (Table 4). In addition, fluid and electrolyte disorder, neurologic disorder, and previous hospital admissions within the past 28 days were independently associated with infection. Low baseline platelet levels approached but did not reach significance (*P* = 0.058); however, this variable was retained, as it significantly improved the performance of the model (*P* = 0.046 for the likelihood ratio test) without significantly altering the other variables’ estimates. The area under the receiver-operator characteristic (AUROC) demonstrated acceptable performance of the model (0.78; 95% CI, 0.718 to 0.842) (Fig. 2), and the Hosmer-Lemeshow test did not indicate poor model fit (*P* = 0.135).

**TABLE 4:** Multiple logistic regression model of risk factors for infection

Variable	OR	95% CI	*P* value
Colonized	4.01	2.08–7.73	<0.001
Fluid and electrolyte disorder^a^	2.37	1.22–4.59	0.011
Neurologic disorder^a^	3.31	1.28–8.54	0.013
Prior admit (28 days)	2.16	1.04–4.48	0.038
Baseline platelet count/100 units (×10^3^/µl)^b^	0.73	0.53–1.01	0.058

a As defined by the Elixhauser index (35).

b For every 100-unit increase in baseline platelet count, the odds of infection was 0.73-fold lower.

### Concordance of colonizing and infecting isolate pairs based on molecular strain typing.

**FIG 3:**
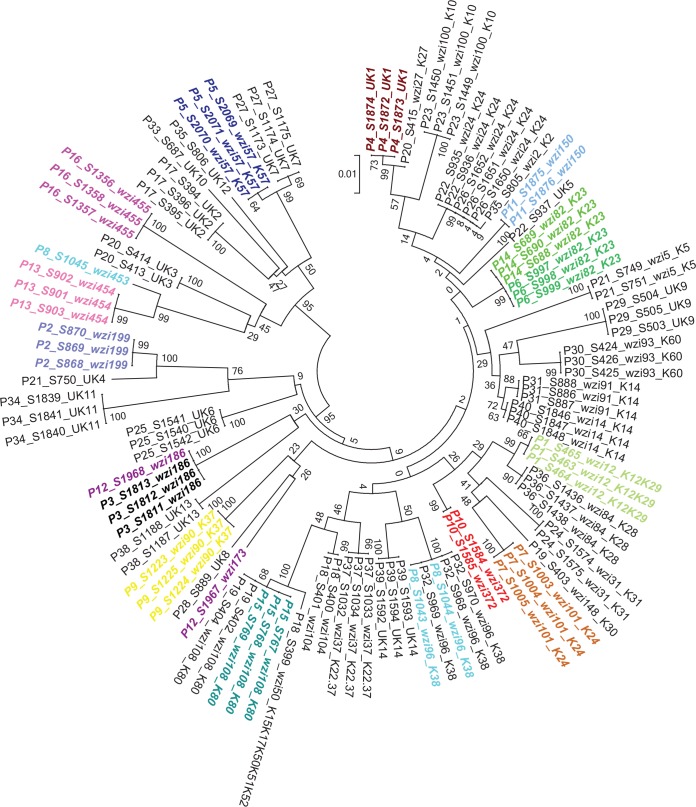
Phylogenetic tree for *wzi* sequence of patient rectal swab isolates. Unique patients (P) are numbered (P1 to P40). A rectal swab (S) isolate is indicated after the patient number and immediately before the isolate number (e.g., S463 is stool isolate number 463). The isolate *wzi* type is indicated, and novel alleles are designated unknown by UK. A total of 110 rectal swab isolates from 40 unique patients were tested for strain type using *wzi* gene sequencing. A total of 43 different *wzi* types of strains were identified. Rectal swab isolates for patients with *K. pneumoniae* colonization prior to infection were all included in the analysis (P1 to P16; colored font). The scale bar represents the amount of genetic change; 0.01 equals 1 change per 100 nucleotide sites. The numbers next to each node are the percentage of iterations that recovered the same node.

To determine if patients become infected with strains with which they were previously colonized, we first screened for genetic differences using *wzi* gene sequencing. Preliminary results from 17 patients indicated that *wzi* sequencing had a similar discriminatory power to MLST, with both distinguishing 16 sequence types among 20 isolates (see Fig. S1 in the supplemental material). In order to assess the diversity of strains with which patients were colonized, we determined the *wzi* types of colonizing isolates from 40 patients. Sixteen of these colonized patients had subsequent positive clinical cultures and met case definitions for BSI, pneumonia, or UTI; 24 patients did not. A total of 110 rectal swab isolates were tested; up to three isolates were obtained from each patient. From these 40 patients, only 8 patients had two *wzi* types detected, and no patients had three types within a sample. Despite the homogeneity within individual patients, 43 different *wzi* types were identified among these 40 patients, suggesting high genetic diversity of colonizing *K. pneumoniae* in this patient population (Fig. 3).

10.1128/mSphere.00261-16.2 Figure S1  The *wzi* sequencing method has similar discriminatory power as MLST. Phylogenetic trees based on MLST results (a) and *wzi* sequencing results (b) are shown, each distinguishing 16 sequence types among 20 *K. pneumoniae* isolates. The scale bar represents the amount of genetic change. The numbers next to each node are the percentages of iterations that recovered the same node. Download Figure S1, TIF file, 0.2 MB. Copyright © 2016 Martin et al. 2016 Martin et al. This content is distributed under the terms of the Creative Commons Attribution 4.0 International license.

Of 21 colonized patients who developed infection, 16 sets of colonizing and infecting isolates were available for analysis. Two out of five patients with BSI (40%) had concordant pairs based on *wzi* sequencing of blood and rectal swab isolates. Respiratory and rectal swab isolates from patients with pneumonia (*n* = 7) demonstrated perfect concordance (7/7) (see Fig. S2 in the supplemental material). Although two patients with pneumonia were each colonized with 2 different *wzi* types, one was concordant with each patient’s respiratory isolate (stool isolate 1043 matched respiratory isolates 733 and 734, and stool isolate 1967 matched respiratory isolate 2005) (see Fig. S2). Analysis of urine and rectal swab isolates from patients with UTI (*n* = 4) also demonstrated perfect concordance (4/4).

10.1128/mSphere.00261-16.3 Figure S2  Phylogenetic trees of rectal swab and infecting isolates based on *wzi* gene sequencing. Phylogenetic trees, built using the neighbor-joining method, for infecting and colonizing isolates from patients with BSI (a), pneumonia (PNA) (b), and UTI (c) are shown along with the fraction of patients with a concordant colonizing-infecting isolate pair. Unique patients are indicated by different colors and labeled as follows: patient number_ isolate number_wzi allele (unless a novel allele), _K-type (if known), where the isolate number prefixes indicate rectal swab (S), blood (B), respiratory (R), or urine (U). The scale bar represents the amount of genetic change. The numbers next to each node are the percentages of iterations that recovered the same node. Download Figure S2, PDF file, 1.1 MB. Copyright © 2016 Martin et al. 2016 Martin et al. This content is distributed under the terms of the Creative Commons Attribution 4.0 International license.

**FIG 4:**
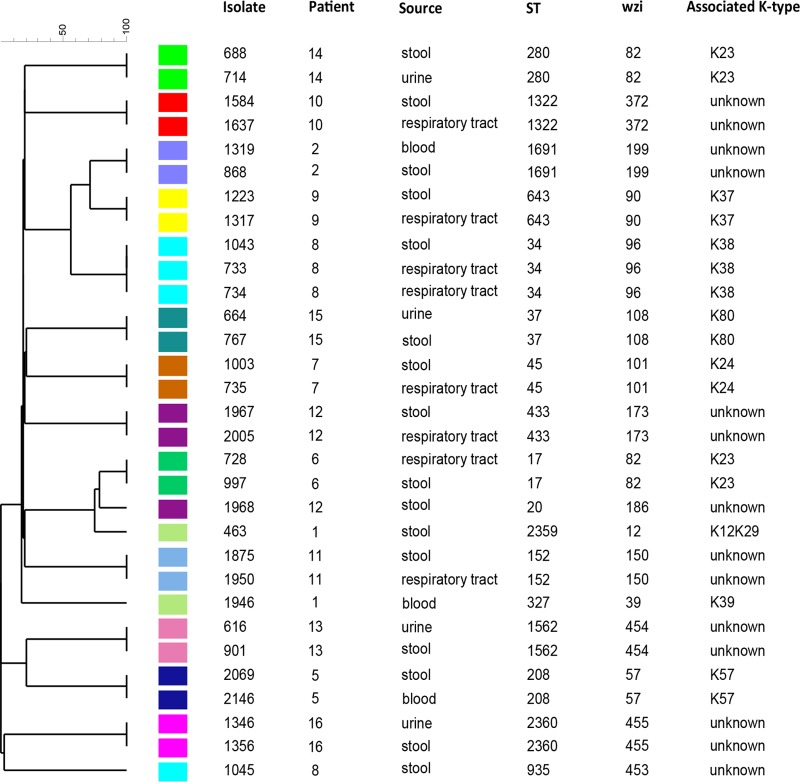
Core genome similarity between infecting and colonizing strains within patients. Patients who had concordant colonizing and infecting isolates based on *wzi* sequencing were further analyzed by WGS and core genome MLST and are represented by a unweighted pair group method using average linkages dendrogram along with isolate number, MLST type (ST), *wzi* type, and body site of culture (source). Each color represents an individual patient. Isolates 463 and 1946 (patient 1) were discordant by *wzi* and were included as a control.

Despite high concordance of colonizing and infecting isolate pairs by *wzi* sequencing, using a single gene typing method may not be sufficient to determine true isolate concordance. To confirm that isolate pairs were the same strain, we performed WGS and determined isolate ST by both 7-gene MLST and 634-gene core genome multilocus sequence typing (cgMLST). We analyzed 13 preliminarily concordant pairs and one unmatched pair as a discordant control (pair 463/1946). MLST analysis showed perfect agreement with *wzi* sequence typing results in identifying 13 concordant pairs (Fig. 4). Two novel STs were identified, ST2359 and ST2360. For patients in whom one of two colonizing isolates matched the infecting isolate, MLST distinguished between the colonizing isolates and indicated that only one was concordant with the infecting isolate.

The cgMLST method provides a more discriminatory approach to defining concordance, since it is based on allelic similarities of 634 *K. pneumoniae* genes (24). All concordant pairs, based on *wzi* and MLST analyses, also clustered together based on cgMLST (Fig. 4). The only pairs that did not group together were our discordant control, and the discordant colonizing isolates (numbers 1045 and 1968) from patients with another colonizing strain that matched the pneumonia isolates. These isolates were also discordant by *wzi* and MLST analyses. To measure the strength of cgMLST concordance of colonizing-infecting pairs within patients compared to between patients, a minimum spanning tree (see Fig. S3 in the supplemental material) was also generated based on the cgMLST data. For example, there were two allelic differences between stool isolate 1223 and pneumonia isolate 1317 in the same patient, while there were 189 allelic differences between these isolates and their closest neighbors from a different patient (pair 1319/868). Overall, there was an average of 2 allelic mismatches between concordant pairs (range, 0 to 7) and 449 allelic mismatches between patients (range, 189 to 629). Taken together, the *wzi*, MLST, and cgMLST data indicate that 100% of urinary and pneumonia isolates tested corresponded to the previously colonizing strain of *K. pneumoniae*.

10.1128/mSphere.00261-16.4 Figure S3  Concordant colonizing-infecting isolate pairs show high core genome allelic similarity. The minimum-spanning tree based on the core genome MLST profiles, onto which the number of allelic differences between isolates, is indicated along the links. Note that the numbers are not additive, and that the tree should not be interpreted as depicting phylogenetic relationships. Isolate names are shown in bold. Each color represents an individual patient. Isolates 463 and 1946 (patient 1) were discordant by *wzi* and included as a control. cgMLST profiles of isolates within a single circle were totally identical. Download Figure S3, PDF file, 0.6 MB. Copyright © 2016 Martin et al. 2016 Martin et al. This content is distributed under the terms of the Creative Commons Attribution 4.0 International license.

### Categorical agreement of antimicrobial susceptibility of colonizing and infecting isolate pairs.

To measure antibiotic susceptibility agreement between concordant and discordant colonizing-infecting isolate pairs, we tested 17 antimicrobials active against Gram-negative bacteria and measured categorical agreement (CA) for susceptible (S), intermediate (I), or resistant (R) isolates based on MIC breakpoints (see Table S1 in the supplemental material) (25). CA was greater than 90% for all antimicrobials tested. However, this isolate collection had a low prevalence of antibiotic resistance. In 12 of 16 patients, both isolates were susceptible to all antimicrobials tested, including the 3 patients with discordant pairs based on sequence type. Two patients with concordant strain types had initially discordant susceptibility results. Only one discrepancy was reproducible by broth microdilution (trimethoprim-sulfamethoxazole in pair 1967/2005). The remaining 11 patients with concordant isolate pairs by sequence type had identical susceptibility patterns, including one ESBL *K. pneumoniae* isolate (pair 767/664).

10.1128/mSphere.00261-16.1 Table S1  Categorical agreement of colonizing-infecting isolate pairs in case patients. Download Table S1, DOCX file, 0.02 MB. Copyright © 2016 Martin et al. 2016 Martin et al. This content is distributed under the terms of the Creative Commons Attribution 4.0 International license.

## DISCUSSION

The objective of this study was to examine the association between *K. pneumoniae* rectal colonization and subsequent extraintestinal *K. pneumoniae* infection. Based on data from 1,765 intensive care and hematology/oncology patients, we found that approximately 1 in 4 patients were rectal carriers of *K. pneumoniae*. We also observed a significant association between rectal *K. pneumoniae* colonization and subsequent infection, even after adjusting for patient variables. Furthermore, there was high concordance among colonizing isolates and subsequent infecting isolates, particularly for pneumonia and UTI, as measured by *wzi*, MLST, and cgMLST analyses. Taken together, these results implicate colonization as a critical step in the pathogenesis of hospital-acquired infections. These results also identify a possible window for intervention to decolonize patients or characterize their colonizing strain in order to predict risk of disease and inform empirical therapy if infection develops.

Our study has several strengths. First, whereas previous studies focused on drug-resistant *K. pneumoniae* or strains involved in outbreaks (26–28), we tested all isolates during a 3-month collection period across multiple wards and units in the hospital. This approach provided comprehensive information on *K. pneumoniae* colonization in the hospital setting and minimized potential selection bias. The large sample size (*n* = 1,765) provided sufficient power to examine the relationship between colonization and patients that met strict case definitions of infection. Second, we used *wzi* gene sequencing to rapidly screen for genetic differences between *K. pneumoniae* isolates (20). We then confirmed concordant pairs by using WGS-based MLST and cgMLST. In a hospital laboratory setting, *wzi* could be used to screen for a potential outbreak strain as a triage step before more costly WGS (20). Lastly, molecular strain typing indicated high *K. pneumoniae* strain diversity in our study population. Molecular epidemiology studies show clonal spread of carbapenem-resistant *K. pneumoniae* in the United States (28, 29). If a dominant clone existed in our population, it would obscure the true association between colonization and subsequent infection. In our diverse setting, the high concordance between colonizing and infecting strains suggests a robust pathogenic mechanism in which patients become infected by their colonizing strain.

This study also has some limitations. First, 35 patients with a positive clinical culture had unknown colonization status prior to the culture date and, thus, were excluded. By excluding this subset of data, we potentially lost cases of infection, and we cannot predict in which direction this would bias the results. Although we collected three rectal swab isolates per patient, most extraintestinal *K. pneumoniae* isolates provided by the clinical lab represented one isolate per site. It is possible that multiple strains may be present at an extraintestinal site but only one isolate was sampled. For *wzi* sequencing, rectal swab isolates from fewer than 10% of colonized patients were tested. Given 40 unique patients with 43 unique strains, almost every patient was colonized with a different strain. This high level of diversity is unlikely to be maintained in the larger sample set. A limitation of the susceptibility data was that the majority of isolates had no detectable acquired resistance. With a low diversity of resistance phenotypes, we were unable to rigorously test the agreement of susceptibility testing between colonizing isolates and subsequent infecting isolates in the same patient. Future studies should determine if high categorical agreement holds in a larger, more resistant colonized-infected patient population.

We conclude, based on three distinct methods, that there is high concordance between colonizing and infecting isolates, particularly for pneumonia and UTI. The discordance in bloodstream infections could be due to exogenous sources of *K. pneumoniae*, such as insertion of an intravenous catheter or a healthcare worker’s hands. The perfect concordance for UTI is consistent with the paradigm for *Escherichia coli* UTI, where fecal colonizing strains contaminate the urogenital tract (30). However, the perfect concordance between rectal isolates and pneumonia isolates was striking. This may indicate simultaneous colonization of the respiratory tract at the time of intestinal acquisition of the strain. This strong concordance suggests that infection prevention approaches or guidance of empirical therapies based on detection and characterization of colonizing *K. pneumoniae* isolates is feasible.

In addition to prior colonization, prior admission, low baseline platelets, and comorbidities of neurologic and fluid and electrolyte disorders were highly predictive of *K. pneumoniae* infection in a multivariable model. The association between healthcare exposure and subsequent infection is plausible, even after adjustment for colonization, since it likely indicates overall poor health status, itself a risk factor for infection. This is likely also true of the other comorbidities included in the final model. The components of this model include information readily available at admission as part of routine testing and chart review. If validated in an independent cohort, rectal screening paired with these variables could rapidly predict risk of *K. pneumoniae* infection.

The finding that patients often become infected with their colonizing strain has strong implications for both infection control and patient care interventions. A recent study in a long-term acute care hospital (LTACH) determined that interventions based on screening for KPC decreased both the colonization rate of patients as well as the rate of clinical infections (31). Moreover, characterization of colonizing strains could inform treatment decisions. Understanding the pathogenic mechanisms of progression from *K. pneumoniae* colonization to disease could enable novel diagnostics and therapeutics to prevent and rapidly treat these common nosocomial infections.

## MATERIALS AND METHODS

### Patient population and setting.

The study was conducted at the University of Michigan Health System (UMHS), a tertiary care hospital with more than 1,000 beds, in Ann Arbor, MI. Approval for this study was granted by the Institutional Review Board of the University of Michigan Medical School. During a 3-month period from 30 July to 31 October 2014, rectal swabs from 1,800 adult (≥18 years old) patients from the intensive care unit (ICU) or hematology/oncology wards were screened for *K. pneumoniae*. Concurrently, extraintestinal *K. pneumoniae* isolates were obtained from the clinical microbiology lab. A total of 1,765 patients had either a rectal swab performed prior to a positive clinical culture or a rectal swab and no positive clinical culture, and these patients were included for analysis of the association between colonization and subsequent infection. Patient demographic characteristics and clinical information was obtained through the electronic medical record (EMR).

### Bacterial identification and growth conditions.

Rectal swabs were collected during the course of clinical care (upon unit admission, weekly, and at discharge) and were transported and stored in an ESwab transport system (BD, Franklin Lakes, NJ) at room temperature. Within 24 h of receipt, 1 µl of inoculated ESwab media was plated onto MacConkey agar (Remel, Lenexa, KS), streaked for quantification, and incubated for 18 to 24 h at 35°C. To ensure collection of the dominant clone in each sample, three mucoid lactose-fermenting (MLF) colonies were isolated as potential *K. pneumoniae* and subcultured onto blood agar plates (BAP; Remel, Lenexa, KS) (32). If fewer than three MLF colonies were present in a particular sample, all were subcultured. Bacterial identification was performed using matrix-assisted laser desorption ionization–time of flight (MALDI-TOF) analysis. Isolates were stored at −80°C in Luria-Bertani (LB) broth containing 20% glycerol and were grown on either BAP or LB plates at 30°C overnight unless otherwise indicated.

### Definitions.

In patients without *K. pneumoniae* infection, *K. pneumoniae* colonization was defined as a positive rectal swab culture for *K. pneumoniae* at any point during the hospital admission. For patients with *K. pneumoniae* infection, colonization was only considered positive if detected prior to the date of the documented infection. If a patient was identified as colonized on more than one date of collection, we used the first positive sample before infection for wzi sequencing, MLST, cgMLST, and antimicrobial susceptibility testing. Patient EMRs were reviewed for any positive culture for *K. pneumoniae* within 90 days of rectal swab culture. Bloodstream infection was defined as any positive blood culture for *K. pneumoniae*. Pneumonia was defined based on a positive *K. pneumoniae* respiratory culture and other Infectious Diseases Society of America (IDSA) diagnostic criteria (33). Patients with positive urine cultures were identified as cases based on the CDC National Healthcare Safety Network (NHSN) UTI case definitions (34). Patients not meeting a case definition of infection within 90 days of a rectal swab culture were considered uninfected. Comorbid disease definitions were extracted from the EMR based on ICD-9 and DRG codes as specified in the Elixhauser index (35). To ensure that we were capturing antibiotic exposure as a risk factor and not an outcome, this variable was constructed to ensure that exposure was present prior to colonization/infection. Exposure to an antibiotic was defined as true for patients without *K. pneumoniae* infection or colonization if administered at any point during the admission. For those patients with colonization, the antibiotic exposure variable was true if antibiotics were started at least 48 hours prior to the detected colonization. For those patients with infection but no preceding colonization, antibiotic exposure was positive if started at least 48 hours prior to documented infection.

### *wzi* gene sequencing.

DNA preparation and PCR amplification were performed as described by Brisse et al. (20) with the following modifications: PCR products were diluted 1:20 in sterile water, and sequencing primers (wzi_for2 and wzi_rev) were diluted to 1 pM/µl prior to submission for Sanger sequencing. Forward and reverse chromatograms were assembled using Lasergene SeqMan (DNASTAR, Madison, WI). Complete alignment was done using ClustalX 2.1 (36). Initial phylogenetic trees were constructed using MEGA 6 (37) based on the neighbor-joining method (500 bootstrap replicates) and Jukes-Cantor distance. The *wzi* library obtained from Brisse et al. (20) was used as a reference in the current analysis.

### Whole-genome sequencing and assembly.

Bacterial genomic DNA (gDNA) was purified using the UltraClean microbial DNA isolation kit (MoBio Laboratories, Inc., Carlsbad, CA). Purified gDNA was sent to the University of Michigan DNA Sequencing Core, where it was sheared (320 bp) and prepared as a multiplex library with unique bar codes for each sample. Whole-genome sequencing was performed using the HiSeq 4000 sequencing system (Illumina, San Diego, CA). Reads were preprocessed for each sample by trimming bases at both ends if the quality score was below 10, using Trimmomatic (v0.32) (38), removing read duplicates using FastUniq (39), and performing error correction using SOAPec (v2.01) (40). Preprocessed reads were assembled using VelvetOptimiser (v2.2.5). In this process, the reads were assembled by using Velvet (41) and a stepwise Kmer size at a step of 2, from 51 to 149 (for paired-end samples), or from 25 to 51 (for shared-end samples). The assembly with the largest *N*_50_ value was used for subsequent analysis.

### MLST and cgMLST.

The gene sequences corresponding to the international MLST scheme of Institut Pasteur (19) were extracted from the genomic assemblies by using the BIGSdb platform (42) and the BLASTN algorithm, and the corresponding allelic number was defined by comparison with the reference nomenclature database (http://bigsdb.pasteur.fr/klebsiella). cgMLST was performed in the same way using the strict core genome MLST scheme defined by Bialek-Davenet et al. (24). Novel alleles and MLST profiles were submitted to the nomenclature database. MLST profiles were compared using the categorical mismatch method within BioNumerics version 6.6 (Applied-Maths, Sint-Martens Latem, Belgium). Uncalled alleles were not considered mismatches in pairwise profile comparisons.

### Antimicrobial susceptibility testing.

Antimicrobial susceptibility testing was performed on the Vitek 2 automated system (bioMérieux, Marcy-l’Étoile, France), using AST-GN82 cards loaded per the manufacturer’s instructions. Isolates were grown on BAP at 37°C overnight. Colonizing and infecting isolates from the same patient were tested in the same batch. Susceptibility testing was performed on one rectal isolate for each patient. ESBL phenotypes were determined with the use of the Vitek 2 Advanced Expert System (AES). Pairs with discrepant results for any antibiotic were tested by Sensititre broth microdilution (Trek Diagnostics Systems, Oakwood Village, OH).

### Statistical analyses.

Initial tests included examination of variables for out-of-range values, measures of central tendency/spread for continuous variables, and frequencies for categorical variables. These initial analyses assisted in constructing variables, including transformations (for example, length of stay was log transformed prior to analysis, given the nonnormal distribution). Initial bivariable analyses were conducted with Student’s *t* test for continuous variables and the chi-square or Fisher’s exact test for categorical variables. Based on these initial analyses, variables with a *P* value of <0.2 on bivariable tests were eligible for inclusion in the final multiple logistic regression model. This final model of *K. pneumoniae* infection was constructed via backwards elimination using a likelihood ratio test for variable retention, with a cutoff α of 0.05. Interactions between variables in the final model were tested and included if significant. Additional model regression assessments included the Hosmer-Lemeshow test for goodness of fit and calculation of the AUROC curve. For interpretation of the results, a *P* value of 0.05 was considered statistically significant for all analyses. The analyses were performed using SAS 9.3 (SAS Institute, Cary, NC) and R 3.2.2 (R Foundation for Statistical Computing, Vienna, Austria).

### Accession number(s).

Whole-genome sequencing files have been deposited in the NCBI Sequence Read Archive (PRJNA341404) under accession numbers SAMN05722982, SAMN05722983, SAMN05722984, SAMN05722985, SAMN05722986, SAMN05722987, SAMN05722988, SAMN05722989, SAMN05722990, SAMN05722991, SAMN05722992, SAMN05722993, SAMN05722994, SAMN05722995, SAMN05722996, SAMN05722997, SAMN05722998, SAMN05722999, SAMN05723000, SAMN05723001, SAMN05723002, SAMN05723003, SAMN05723004, SAMN05723005, SAMN05723006, SAMN05723007, SAMN05723008, SAMN05723009, SAMN05723010, SAMN05723011, and SAMN05723012.

Sequences of *wzi* alleles used for strain comparison are included in Text S1 in the supplemental material. All other data are available upon request.

10.1128/mSphere.00261-16.5 Text S1  *wzi* alleles used to construct phylogenetic trees. Download Text S1, DOC file, 0.1 MB. Copyright © 2016 Martin et al. 2016 Martin et al. This content is distributed under the terms of the Creative Commons Attribution 4.0 International license.

## References

[1] Magill SS, Edwards JR, Bamberg W, Beldavs ZG, Dumyati G, Kainer MA, Lynfield R, Maloney M, McAllister-Hollod L, Nadle J, Ray SM, Thompson DL, Wilson LE, Fridkin SK 2014 Multistate point-prevalence survey of health care-associated infections. N Engl J Med 370:1198–1208. doi:10.1056/NEJMoa1306801. 24670166 PMC4648343

[2] Podschun R, Ullmann U 1998 *Klebsiella* spp. as nosocomial pathogens: epidemiology, taxonomy, typing methods, and pathogenicity factors. Clin Microbiol Rev 11:589–603. 9767057 10.1128/cmr.11.4.589 PMC88898

[3] Paterson DL, Bonomo RA 2005 Extended-spectrum β-lactamases: a clinical update. Clin Microbiol Rev 18:657–686. doi:10.1128/CMR.18.4.657-686.2005. 16223952 PMC1265908

[4] Arnold RS, Thom KA, Sharma S, Phillips M, Kristie Johnson J, Morgan DJ 2011 Emergence of *Klebsiella pneumoniae* carbapenemase (KPC)-producing bacteria. South Med J 104:40–45. doi:10.1097/SMJ.0b013e3181fd7d5a. 21119555 PMC3075864

[5] CDC 2014 Antibiotic resistance threats in the United States, 2013. Centers for Disease Control and Prevention, Atlanta, GA.

[6] Guh AY, Bulens SN, Mu Y, Jacob JT, Reno J, Scott J, Wilson LE, Vaeth E, Lynfield R, Shaw KM, Vagnone PM, Bamberg WM, Janelle SJ, Dumyati G, Concannon C, Beldavs Z, Cunningham M, Cassidy PM, Phipps EC, Kenslow N 2015 Epidemiology of carbapenem-resistant *Enterobacteriaceae* in 7 US communities, 2012-2013. JAMA 314:1479–1487. doi:10.1001/jama.2015.12480. 26436831 PMC6492240

[7] Selden R, Lee S, Wang WL, Bennett JV, Eickhoff TC 1971 Nosocomial *Klebsiella* infections: intestinal colonization as a reservoir. Ann Intern Med 74:657–664. doi:10.7326/0003-4819-74-5-657. 5559431

[8] Jarvis WR, Munn VP, Highsmith AK, Culver DH, Hughes JM 1985 The epidemiology of nosocomial infections caused by *Klebsiella pneumoniae*. Infect Control 6:68–74. doi:10.1017/S0195941700062639. 3882593

[9] Casewell M, Phillips I 1977 Hands as route of transmission for *Klebsiella* species. Br Med J 2:1315–1317. doi:10.1136/bmj.2.6098.1315. 589166 PMC1632544

[10] Filius PM, Gyssens IC, Kershof IM, Roovers PJ, Ott A, Vulto AG, Verbrugh HA, Endtz HP 2005 Colonization and resistance dynamics of gram-negative bacteria in patients during and after hospitalization. Antimicrob Agents Chemother 49:2879–2886. doi:10.1128/AAC.49.7.2879-2886.2005. 15980364 PMC1168677

[11] Rose HD, Babcock JB 1975 Colonization of intensive care unit patients with gram-negative bacilli. Am J Epidemiol 101:495–501. 1098452 10.1093/oxfordjournals.aje.a112120

[12] Chung DR, Lee H, Park MH, Jung SI, Chang HH, Kim YS, Son JS, Moon C, Kwon KT, Ryu SY, Shin SY, Ko KS, Kang CI, Peck KR, Song JH 2012 Fecal carriage of serotype K1 *Klebsiella pneumoniae* ST23 strains closely related to liver abscess isolates in Koreans living in Korea. Eur J Clin Microbiol Infect Dis 31:481–486. doi:10.1007/s10096-011-1334-7. 21739348

[13] Pollack M, Nieman R, Reinhardt J, Charache P, Jett M, Hardy P 1972 Factors influencing colonisation and antibiotic-resistance patterns of gram-negative bacteria in hospital patients. Lancet 300:668–671. doi:10.1016/S0140-6736(72)92084-3. 4115815

[14] Asensio A, Oliver A, González-Diego P, Baquero F, Pérez-Díaz JC, Ros P, Cobo J, Palacios M, Lasheras D, Cantón R 2000 Outbreak of a multiresistant *Klebsiella pneumoniae* strain in an intensive care unit: antibiotic use as risk factor for colonization and infection. Clin Infect Dis 30:55–60. doi:10.1086/313590. 10619733

[15] Snitkin ES, Zelazny AM, Thomas PJ, Stock F, NISC Comparative Sequencing Program Group, Henderson DK, Palmore TN, Segre JA 2012 Tracking a hospital outbreak of carbapenem-resistant *Klebsiella pneumoniae* with whole-genome sequencing. Sci Transl Med 4:148ra116. doi:10.1126/scitranslmed.3004129. PMC3521604 22914622

[16] Hasan CM, Turlej-Rogacka A, Vatopoulos AC, Giakkoupi P, Maâtallah M, Giske CG 2014 Dissemination of *bla*_VIM_ in Greece at the peak of the epidemic of 2005-2006: clonal expansion of *Klebsiella pneumoniae* clonal complex 147. Clin Microbiol Infect 20:34–37. doi:10.1111/1469-0691.12187. 23464880

[17] Van Duin D, Cober E, Richter SS, Perez F, Kalayjian RC, Salata RA, Evans S, Fowler VG Jr, Kaye KS, Bonomo RA 2015 Impact of therapy and strain type on outcomes in urinary tract infections caused by carbapenem-resistant *Klebsiella pneumoniae*. J Antimicrob Chemother 70:1203–1211. doi:10.1093/jac/dku495. 25492391 PMC4356203

[18] Viau RA, Hujer AM, Marshall SH, Perez F, Hujer KM, Briceño DF, Dul M, Jacobs MR, Grossberg R, Toltzis P, Bonomo RA 2012 “Silent” dissemination of *Klebsiella pneumoniae* isolates bearing *K. pneumoniae* carbapenemase in a long-term care facility for children and young adults in northeast Ohio. Clin Infect Dis 54:1314–1321. doi:10.1093/cid/cis036. 22492318 PMC3404693

[19] Diancourt L, Passet V, Verhoef J, Grimont PA, Brisse S 2005 Multilocus sequence typing of *Klebsiella pneumoniae* nosocomial isolates. J Clin Microbiol 43:4178–4182. doi:10.1128/JCM.43.8.4178-4182.2005. 16081970 PMC1233940

[20] Brisse S, Passet V, Haugaard AB, Babosan A, Kassis-Chikhani N, Struve C, Decré D 2013 wzi gene sequencing, a rapid method for determination of capsular type for *Klebsiella* strains. J Clin Microbiol 51:4073–4078. doi:10.1128/JCM.01924-13. 24088853 PMC3838100

[21] Diago-Navarro E, Chen L, Passet V, Burack S, Ulacia-Hernando A, Kodiyanplakkal RP, Levi MH, Brisse S, Kreiswirth BN, Fries BC 2014 Carbapenem-resistant *Klebsiella pneumoniae* exhibit variability in capsular polysaccharide and capsule associated virulence traits. J Infect Dis 210:803–813. doi:10.1093/infdis/jiu157. 24634498 PMC4432395

[22] Cubero M, Grau I, Tubau F, Pallarés R, Dominguez MA, Liñares J, Ardanuy C 2016 Hypervirulent *Klebsiella pneumoniae* clones causing bacteraemia in adults in a teaching hospital in Barcelona, Spain (2007-2013). Clin Microbiol Infect 22:154–160. doi:10.1016/j.cmi.2015.09.025. 26454059

[23] Wright MS, Perez F, Brinkac L, Jacobs MR, Kaye K, Cober E, van Duin D, Marshall SH, Hujer AM, Rudin SD, Hujer KM, Bonomo RA, Adams MD 2014 Population structure of KPC-producing *Klebsiella pneumoniae* isolates from midwestern U.S. hospitals. Antimicrob Agents Chemother 58:4961–4965. doi:10.1128/AAC.00125-14. 24913165 PMC4136011

[24] Bialek-Davenet S, Criscuolo A, Ailloud F, Passet V, Jones L, Delannoy-Vieillard AS, Garin B, Le Hello S, Arlet G, Nicolas-Chanoine MH, Decré D, Brisse S 2014 Genomic definition of hypervirulent and multidrug-resistant *Klebsiella pneumoniae* clonal groups. Emerg Infect Dis 20:1812–1820. doi:10.3201/eid2011.140206. 25341126 PMC4214299

[25] CLSI 2015 Performance standards for antimicrobial testing; twenty-fifth informational supplement. Clinical and Laboratory Standards Institute, Wayne, PA.

[26] Tascini C, Lipsky BA, Iacopi E, Ripoli A, Sbrana F, Coppelli A, Goretti C, Piaggesi A, Menichetti F 2015 KPC-producing *Klebsiella pneumoniae* rectal colonization is a risk factor for mortality in patients with diabetic foot infections. Clin Microbiol Infect 21:790.e1–790.e3. doi:10.1016/j.cmi.2015.04.010. 25911991

[27] Dautzenberg MJ, Wekesa AN, Gniadkowski M, Antoniadou A, Giamarellou H, Petrikkos GL, Skiada A, Brun-Buisson C, Bonten MJ, Derde LP, Mastering hOSpital Antimicrobial Resistance in Europe Work Package 3 Study Team 2015 The association between colonization with carbapenemase-producing Enterobacteriaceae and overall ICU mortality: an observational cohort study. Crit Care Med 43:1170–1177. doi:10.1097/CCM.0000000000001028. 25882764 PMC4431676

[28] Kitchel B, Rasheed JK, Patel JB, Srinivasan A, Navon-Venezia S, Carmeli Y, Brolund A, Giske CG 2009 Molecular epidemiology of KPC-producing *Klebsiella pneumoniae* isolates in the United States: clonal expansion of multilocus sequence type 258. Antimicrob Agents Chemother 53:3365–3370. doi:10.1128/AAC.00126-09. 19506063 PMC2715580

[29] Endimiani A, Hujer AM, Perez F, Bethel CR, Hujer KM, Kroeger J, Oethinger M, Paterson DL, Adams MD, Jacobs MR, Diekema DJ, Hall GS, Jenkins SG, Rice LB, Tenover FC, Bonomo RA 2009 Characterization of *bla*_KPC_-containing *Klebsiella pneumoniae* isolates detected in different institutions in the eastern USA. J Antimicrob Chemother 63:427–437. doi:10.1093/jac/dkn547. 19155227 PMC2640158

[30] Henderson JP, Crowley JR, Pinkner JS, Walker JN, Tsukayama P, Stamm WE, Hooton TM, Hultgren SJ 2009 Quantitative metabolomics reveals an epigenetic blueprint for iron acquisition in uropathogenic *Escherichia coli*. PLoS Pathog 5:e1000305. doi:10.1371/journal.ppat.1000305. 19229321 PMC2637984

[31] Hayden MK, Lin MY, Lolans K, Weiner S, Blom D, Moore NM, Fogg L, Henry D, Lyles R, Thurlow C, Sikka M, Hines D, Weinstein RA, Centers for Disease Control and Prevention Epicenters Program 2015 Prevention of colonization and infection by *Klebsiella pneumoniae* carbapenemase-producing *Enterobacteriaceae* in long-term acute-care hospitals. Clin Infect Dis 60:1153–1161. doi:10.1093/cid/ciu1173. 25537877 PMC8381216

[32] Lidin-Janson G, Kaijser B, Lincoln K, Olling S, Wedel H 1978 The homogeneity of the faecal coliform flora of normal school-girls, characterized by serological and biochemical properties. Med Microbiol Immunol 164:247–253. doi:10.1007/BF02125493. 45599

[33] ATS 2005 Guidelines for the management of adults with hospital-acquired, ventilator-associated, and healthcare-associated pneumonia. Am J Respir Crit Care Med 171:388–416. doi:10.1164/rccm.200405-644ST. 15699079

[34] CDC 2016 Urinary tract infection (catheter-associated urinary tract infection [CAUTI] and non-catheter-associated urinary tract infection [UTI]) and other urinary system infection [USI]) events. Centers for Disease Control and Prevention, Atlanta, GA.

[35] Elixhauser A, Steiner C, Harris DR, Coffey RM 1998 Comorbidity measures for use with administrative data. Med Care 36:8–27. doi:10.1097/00005650-199801000-00004. 9431328

[36] Larkin MA, Blackshields G, Brown NP, Chenna R, McGettigan PA, McWilliam H, Valentin F, Wallace IM, Wilm A, Lopez R, Thompson JD, Gibson TJ, Higgins DG 2007 Clustal W and Clustal X version 2.0. BioInformatics 23:2947–2948. doi:10.1093/bioinformatics/btm404. 17846036

[37] Tamura K, Stecher G, Peterson D, Filipski A, Kumar S 2013 MEGA6: molecular evolutionary genetics analysis version 6.0. Mol Biol Evol 30:2725–2729 doi:10.1093/molbev/mst197. 24132122 PMC3840312

[38] Bolger AM, Lohse M, Usadel B 2014 Trimmomatic: a flexible trimmer for Illumina sequence data. Bioinformatics 30:2114–2120 doi:10.1093/bioinformatics/btu170. 24695404 PMC4103590

[39] Xu H, Luo X, Qian J, Pang X, Song J, Qian G, Chen J, Chen S 2012 FastUniq: a fast de novo duplicates removal tool for paired short reads. PLoS One 7:e52249. doi:10.1371/journal.pone.0052249. 23284954 PMC3527383

[40] Luo R, Liu B, Xie Y, Li Z, Huang W, Yuan J, He G, Chen Y, Pan Q, Liu Y, Tang J, Wu G, Zhang H, Shi Y, Liu Y, Yu C, Wang B, Lu Y, Han C, Cheung DW, Yiu SM, Peng S, Xiaoqian Z, Liu G, Liao X, Li Y, Yang H, Wang J, Lam TW, Wang J 2012 SOAPdenovo2: an empirically improved memory-efficient short-read de novo assembler. GigaScience 1:18. doi:10.1186/2047-217X-1-18. 23587118 PMC3626529

[41] Zerbino DR, Birney E 2008 Velvet: algorithms for de novo short read assembly using de Bruijn graphs. Genome Res 18:821–829. doi:10.1101/gr.074492.107. 18349386 PMC2336801

[42] Jolley KA, Maiden MC 2010 BIGSdb: scalable analysis of bacterial genome variation at the population level. BMC Bioinformatics 11:595. doi:10.1186/1471-2105-11-595. 21143983 PMC3004885

