# Supplementary figures and images for: Molecular Epidemiology of Colonizing and Infecting Isolates of Klebsiella pneumoniae
Source: mSphere. 2016 Oct 19;1(5):e00261-16. doi: 10.1128/mSphere.00261-16 (PMC5071533; doi:10.1128/mSphere.00261-16)

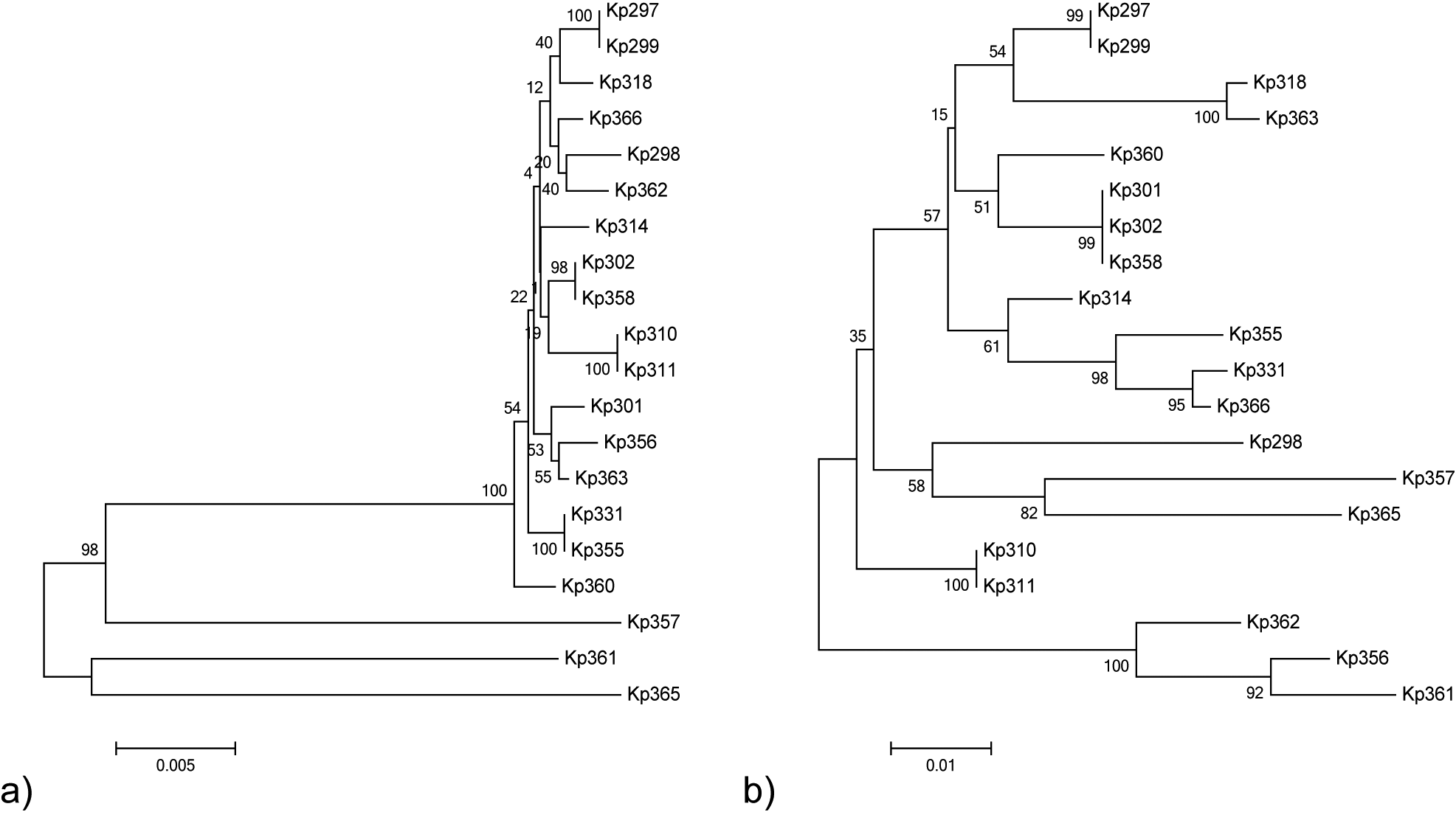

Supplement: Figure S1 [file sph005162166sf2.tif]

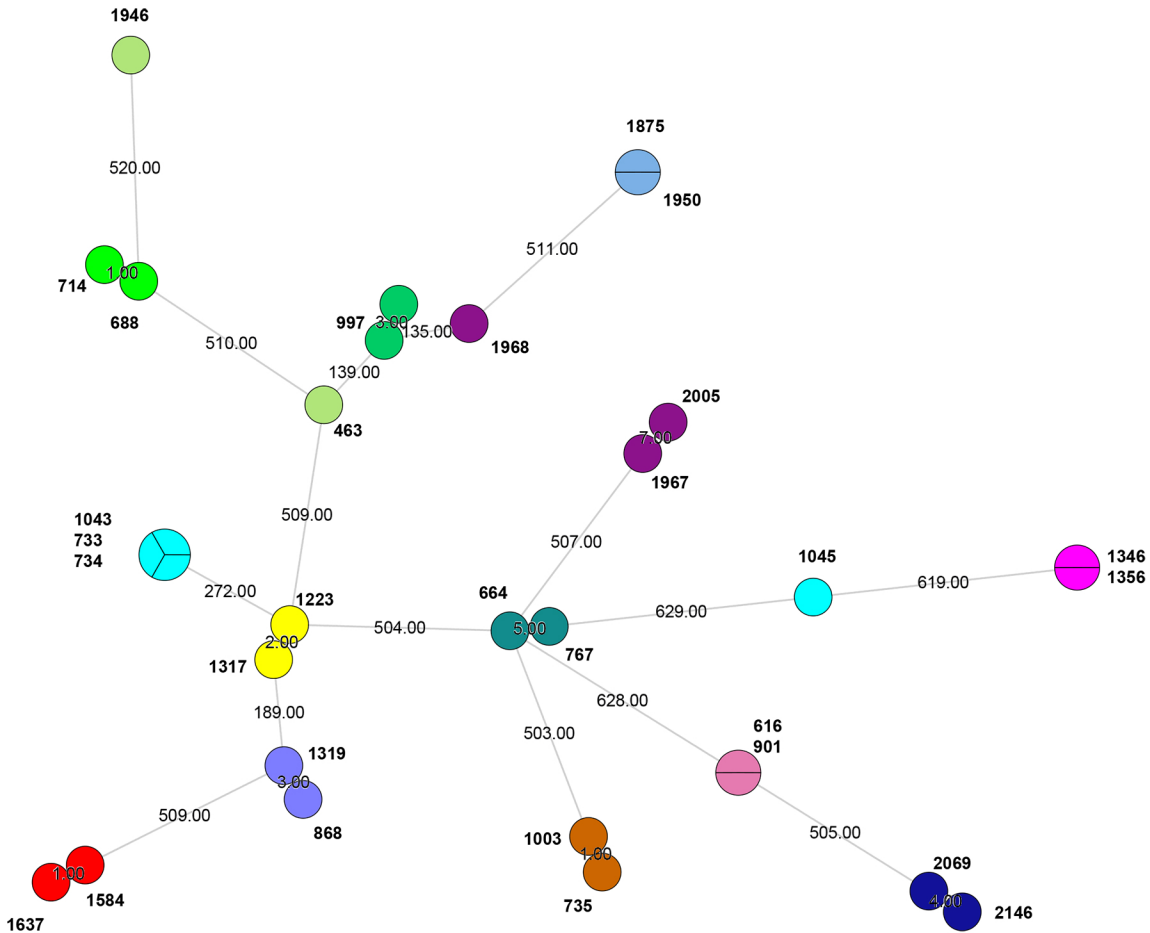

Supplement: Figure S3 [file sph005162166sf4.pdf]
