# Supplementary material for: Molecular Epidemiology of Colonizing and Infecting Isolates of Klebsiella pneumoniae
Source: mSphere. 2016 Oct 19;1(5):e00261-16. doi: 10.1128/mSphere.00261-16 (PMC5071533; doi:10.1128/mSphere.00261-16)
Supplement: Figure S2 [file sph005162166sf3.pdf]

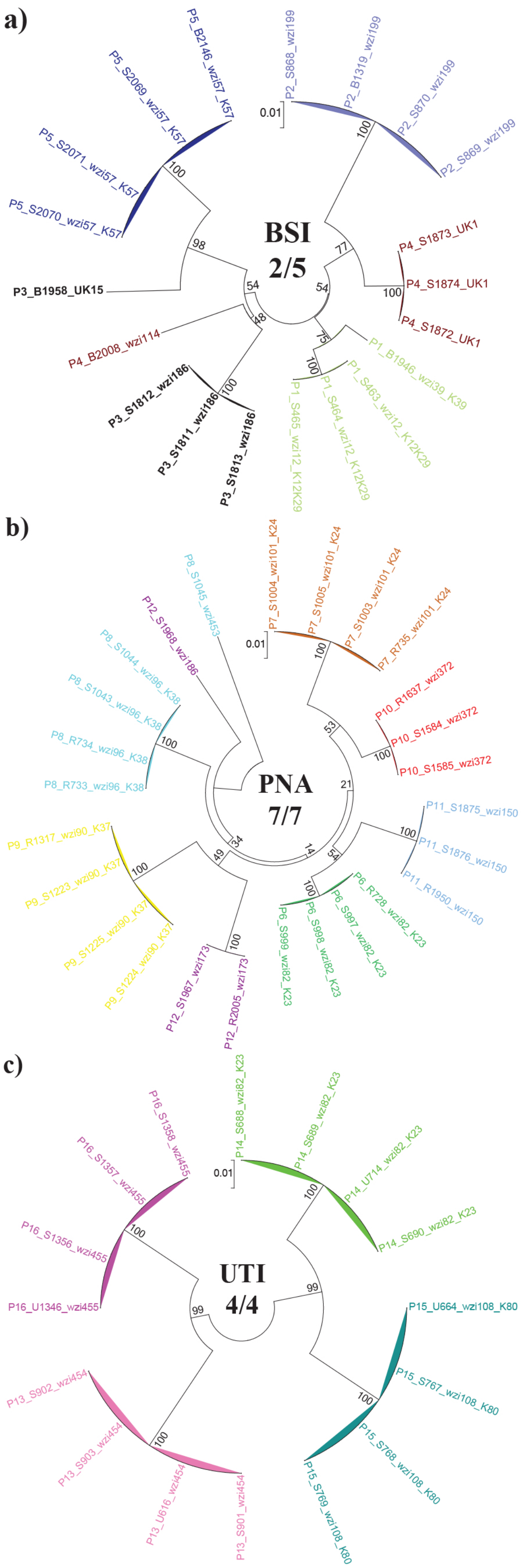
a)
BSI
2/5
0.01
P2_S868_wzi199
P2_B1319_wzi199
P2_S870_wzi199
P2_S869_wzi199
P5_B2146_wzi57_K57
P5_S2069_wzi57_K57
P5_S2071_wzi57_K57
P5_S2070_wzi57_K57
P3_B1958_UK15
P4_S1873_UK1
P4_S1874_UK1
P4_S1872_UK1
P4_B2008_wzi114
P3_S1812_wzi186
P3_S1811_wzi186
P3_S1813_wzi186
P1_B1946_wzi39_K39
P1_S463_wzi12_K12K29
P1_S464_wzi12_K12K29
P1_S465_wzi12_K12K29
100
100
98
77
54
54
48
100
75
100
100
b)
PNA
7/7
0.01
P7_S1004_wzi101_K24
P7_S1005_wzi101_K24
P7_S1003_wzi101_K24
P7_R735_wzi101_K24
P8_S1045_wzi453
P12_S1968_wzi186
P8_S1044_wzi96_K38
P8_S1043_wzi96_K38
P8_R734_wzi96_K38
P8_R733_wzi96_K38
P10_R1637_wzi372
P10_S1584_wzi372
P10_S1585_wzi372
P11_S1875_wzi150
P11_S1876_wzi150
P11_R1950_wzi150
P9_R1317_wzi90_K37
P9_S1223_wzi90_K37
P9_S1225_wzi90_K37
P9_S1224_wzi90_K37
P12_S1967_wzi173
P12_R2005_wzi173
P6_S999_wzi82_K23
P6_S998_wzi82_K23
P6_S997_wzi82_K23
P6_R728_wzi82_K23
100
100
53
100
21
100
100
34
49
14
54
100
100
c)
UTI
4/4
0.01
P14_S688_wzi82_K23
P14_S689_wzi82_K23
P14_U714_wzi82_K23
P14_S690_wzi82_K23
P16_S1358_wzi455
P16_S1357_wzi455
P16_S1356_wzi455
P16_U1346_wzi455
P15_U664_wzi108_K80
P15_S767_wzi108_K80
P15_S768_wzi108_K80
P15_S769_wzi108_K80
P13_S902_wzi454
P13_S903_wzi454
P13_U616_wzi454
P13_S901_wzi454
100
100
99
99
100
100
