# Supplementary material for: Molecular Epidemiology of Colonizing and Infecting Isolates of Klebsiella pneumoniae
Source: mSphere. 2016 Oct 19;1(5):e00261-16. doi: 10.1128/mSphere.00261-16 (PMC5071533; doi:10.1128/mSphere.00261-16)
Supplement: Table S1 [file sph005162166st1.docx]

**Table S1. Categorical agreement of colonizing-infecting isolate pairs in case patients**

| **Antibiotic** | **Categorical result of isolate pair by infection site, patient number, and *wzi* Match (Y/N)^a^** | | | | | | | | | | | | | | | | **Categorical agreement (%)** |
| --- | --- | --- | --- | --- | --- | --- | --- | --- | --- | --- | --- | --- | --- | --- | --- | --- | --- |
|  | **BSI** | | | | | **Pneumonia** | | | | | | | **UTI** | | | |  |
|  | **1** | **2** | **3** | **4** | **5** | **6** | **7^b^** | **8** | **9** | **10** | **11** | **12^c^** | **13** | **14** | **15** | **16** |  |
|  | **N** | **Y** | **N** | **N** | **Y** | **Y** | **Y** | **Y** | **Y** | **Y** | **Y** | **Y** | **Y** | **Y** | **Y** | **Y** |  |
| **Amp/Sul** | S | I | S | S | S | S | R | S | S | S | S | R/S | S | S | R | S | 94 |
| **Pip/Tazo** | S | S | S | S | S | S | I/R | S | S | S | S | S | S | S | R | S | 94 |
| **Cefazolin** | S | S | S | S | S | S | S | S | S | S | S | S | S | S | R | S | 100 |
| **Ceftazidime** | S | S | S | S | S | S | S | S | S | S | S | S | S | S | R | S | 100 |
| **Ceftriaxone** | S | S | S | S | S | S | S | S | S | S | S | S | S | S | R | S | 100 |
| **Cefepime** | S | S | S | S | S | S | S | S | S | S | S | S | S | S | R | S | 100 |
| **Aztreonam** | S | S | S | S | S | S | S | S | S | S | S | S | S | S | R | S | 100 |
| **Ertapenem** | S | S | S | S | S | S | S | S | S | S | S | S | S | S | S | S | 100 |
| **Imipenem** | S | S | S | S | S | S | S | S | S | S | S | S | S | S | S | S | 100 |
| **Meropenem** | S | S | S | S | S | S | S | S | S | S | S | S | S | S | S | S | 100 |
| **Amikacin** | S | S | S | S | S | S | S | S | S | S | S | S | S | S | S | S | 100 |
| **Gentamicin** | S | S | S | S | S | S | S | S | S | S | S | I/S | S | S | S | S | 94 |
| **Tobramycin** | S | S | S | S | S | S | S | S | S | S | S | S | S | S | S | S | 100 |
| **Ciprofloxacin** | S | S | S | S | S | S | S | S | S | S | S | S | S | S | S | S | 100 |
| **Levofloxacin** | S | S | S | S | S | S | S | S | S | S | S | S | S | S | S | S | 100 |
| **Tigecycline** | S | R | S | S | S | S | S/R | S | S | S | S | S | S | S | S | S | 94 |
| **TMP/Sulfa** | S | S | S | S | S | S | S | S | S | S | S | R/S | S | S | R | S | 94 |

^a^ Combined categorical results of Susceptible (S), Intermediate (I), or Resistant (R) for each pair are shown; discrepant results confirmed by broth microdilution are shown as the result for colonizing/infecting isolate and highlighted in red; discrepant results that could not be confirmed are in yellow; concordant non-susceptible results are in green.

^b^ Patient 7 isolates were S/S for both Pip/Tazo and Tigecycline by broth microdilution

^c^ Patient 12 isolates were I/S for Ampicillin/Sulbactam and S/S for Gentamicin by broth microdilution
