# Supplementary material for: Molecular Epidemiology of Colonizing and Infecting Isolates of Klebsiella pneumoniae
Source: mSphere. 2016 Oct 19;1(5):e00261-16. doi: 10.1128/mSphere.00261-16 (PMC5071533; doi:10.1128/mSphere.00261-16)
Supplement: Text S1 [file sph005162166s5.doc]

>P1_B1946_wzi39_K39

ATGATAAAAATTGCGCGCATTGCCGTGACGTTGGGTTTGCTTTCCTCCCTGGGAGCCCAG

GCTTACGCGGCCGGGTTAGTGGTAAATGACAACGACTTGCGTAACGACCTGGCCTGGCTT

TCCGATCGCGGGGTCATCCATCTGAGCCTGTCGACCTGGCCGCTGAGCCAGGAAGAGATC

GCCCGGGCACTGAAAAAGGCCAAACCGTCTTATTCTTCTGAACAAGTGGTGCTGGCGCGT

ATTAACCAGCGACTGTCTGCCTTAAAAGCCGATTTCCGGGTCACCGGCTACACTTCAACC

GATCAGCCGGGCACTCCACAGGGGTTTGGTCAGACACAGCCGGCGGATAACTCGTTAGGC

CTGGCGTTCAACAACAGCGGCGAGTGGTGGGATGTTCACCTGCAGGGCAACGTCGAAGGG

GGGGAGCGGATCAGCAACGGATCGCGC

>P1_S463_wzi12_K12K29

ATGATAAAAATTGCGCGCATTGCCGTGACGTTGGGTTTGCTTTCCTCCCTGGGAGCCCAG

GCTTACGCGGCCGGGTTAGTGGTAAATGACAACGACTTGCGTAACGACCTGGCCTGGCTT

TCCGATCGCGGGGTCATCCATCTGAGCCTGTCGACCTGGCCGCTGAGCCAGGAAGAGATC

GCCCGGGCACTGAAAAAGGCTAAACCGTCCTATTCTTCTGAACAAGTGGTGCTGGCGCGT

ATTAACCAGCGACTGTCTGCCTTAAAAGCGGATTTCCGGGTCACCGGCTACACTTCAACC

GATCAGCCGGGTACTCCGCAGGGGTTTGGCCAGACGCAGCCGGCGGATAACTCGTTAGGC

CTGGCGTTCAACAACAGCGGCGAGTGGTGGGATGTTCACCTGCAGGGCAACGTTGAAGGG

GGAGAGCGGATCAGCAACGGATCGCGC

>P1_S464_wzi12_K12K29

ATGATAAAAATTGCGCGCATTGCCGTGACGTTGGGTTTGCTTTCCTCCCTGGGAGCCCAG

GCTTACGCGGCCGGGTTAGTGGTAAATGACAACGACTTGCGTAACGACCTGGCCTGGCTT

TCCGATCGCGGGGTCATCCATCTGAGCCTGTCGACCTGGCCGCTGAGCCAGGAAGAGATC

GCCCGGGCACTGAAAAAGGCTAAACCGTCCTATTCTTCTGAACAAGTGGTGCTGGCGCGT

ATTAACCAGCGACTGTCTGCCTTAAAAGCGGATTTCCGGGTCACCGGCTACACTTCAACC

GATCAGCCGGGTACTCCGCAGGGGTTTGGCCAGACGCAGCCGGCGGATAACTCGTTAGGC

CTGGCGTTCAACAACAGCGGCGAGTGGTGGGATGTTCACCTGCAGGGCAACGTTGAAGGG

GGAGAGCGGATCAGCAACGGATCGCGC

>P1_S465_wzi12_K12K29

ATGATAAAAATTGCGCGCATTGCCGTGACGTTGGGTTTGCTTTCCTCCCTGGGAGCCCAG

GCTTACGCGGCCGGGTTAGTGGTAAATGACAACGACTTGCGTAACGACCTGGCCTGGCTT

TCCGATCGCGGGGTCATCCATCTGAGCCTGTCGACCTGGCCGCTGAGCCAGGAAGAGATC

GCCCGGGCACTGAAAAAGGCTAAACCGTCCTATTCTTCTGAACAAGTGGTGCTGGCGCGT

ATTAACCAGCGACTGTCTGCCTTAAAAGCGGATTTCCGGGTCACCGGCTACACTTCAACC

GATCAGCCGGGTACTCCGCAGGGGTTTGGCCAGACGCAGCCGGCGGATAACTCGTTAGGC

CTGGCGTTCAACAACAGCGGCGAGTGGTGGGATGTTCACCTGCAGGGCAACGTTGAAGGG

GGAGAGCGGATCAGCAACGGATCGCGC

>P2_B1319_wzi199

ATGATAAAAATTGCGCGCATTGCCGTGACGCTGGGGTTGCTTTCCTCTCTGGGAGCCCAG

GCTTACGCGGCTGGGTTAGTGGTAAATGACAACGATTTGAGAAATGACCTTGCCTGGCTT

TCCGACCGTGGAGTTATTCATCTGAGCCTGTCGACGTGGCCGCTGAGTCAGGAAGAGATC

TCCCGGGCGCTGAAAAAGGCCAAACCTTCCTATTCTTCTGAGCAAGTGGTGCTGGCTCGA

ATTAACCAGCGACTGTCTGCCTTAAAAGCCGATTTTCGGGTCACCGGCTACACTTCAACC

GATCAGCCGGGCACTCCGCAGGGGTTTGGTCAGACACAGCCGGCAGATAACTCGTTAGGC

CTGGCGTTCAACAACAGCGGCGAGTGGTGGGATATCCACCTCCAGGGCAACGTCGAAGGA

GGGGAGCGGATCAGCAACGGATCGCGC

>P2_S868_wzi199

ATGATAAAAATTGCGCGCATTGCCGTGACGCTGGGGTTGCTTTCCTCTCTGGGAGCCCAG

GCTTACGCGGCTGGGTTAGTGGTAAATGACAACGATTTGAGAAATGACCTTGCCTGGCTT

TCCGACCGTGGAGTTATTCATCTGAGCCTGTCGACGTGGCCGCTGAGTCAGGAAGAGATC

TCCCGGGCGCTGAAAAAGGCCAAACCTTCCTATTCTTCTGAGCAAGTGGTGCTGGCTCGA

ATTAACCAGCGACTGTCTGCCTTAAAAGCCGATTTTCGGGTCACCGGCTACACTTCAACC

GATCAGCCGGGCACTCCGCAGGGGTTTGGTCAGACACAGCCGGCAGATAACTCGTTAGGC

CTGGCGTTCAACAACAGCGGCGAGTGGTGGGATATCCACCTCCAGGGCAACGTCGAAGGA

GGGGAGCGGATCAGCAACGGATCGCGC

>P2_S869_wzi199

ATGATAAAAATTGCGCGCATTGCCGTGACGCTGGGGTTGCTTTCCTCTCTGGGAGCCCAG

GCTTACGCGGCTGGGTTAGTGGTAAATGACAACGATTTGAGAAATGACCTTGCCTGGCTT

TCCGACCGTGGAGTTATTCATCTGAGCCTGTCGACGTGGCCGCTGAGTCAGGAAGAGATC

TCCCGGGCGCTGAAAAAGGCCAAACCTTCCTATTCTTCTGAGCAAGTGGTGCTGGCTCGA

ATTAACCAGCGACTGTCTGCCTTAAAAGCCGATTTTCGGGTCACCGGCTACACTTCAACC

GATCAGCCGGGCACTCCGCAGGGGTTTGGTCAGACACAGCCGGCAGATAACTCGTTAGGC

CTGGCGTTCAACAACAGCGGCGAGTGGTGGGATATCCACCTCCAGGGCAACGTCGAAGGA

GGGGAGCGGATCAGCAACGGATCGCGC

>P2_S870_wzi199

ATGATAAAAATTGCGCGCATTGCCGTGACGCTGGGGTTGCTTTCCTCTCTGGGAGCCCAG

GCTTACGCGGCTGGGTTAGTGGTAAATGACAACGATTTGAGAAATGACCTTGCCTGGCTT

TCCGACCGTGGAGTTATTCATCTGAGCCTGTCGACGTGGCCGCTGAGTCAGGAAGAGATC

TCCCGGGCGCTGAAAAAGGCCAAACCTTCCTATTCTTCTGAGCAAGTGGTGCTGGCTCGA

ATTAACCAGCGACTGTCTGCCTTAAAAGCCGATTTTCGGGTCACCGGCTACACTTCAACC

GATCAGCCGGGCACTCCGCAGGGGTTTGGTCAGACACAGCCGGCAGATAACTCGTTAGGC

CTGGCGTTCAACAACAGCGGCGAGTGGTGGGATATCCACCTCCAGGGCAACGTCGAAGGA

GGGGAGCGGATCAGCAACGGATCGCGC

>P3_B1958_UK15

ATGATAAAAATTGCGCGCATTGCCGTGACATTGGGCTTGCTTTCCTCACTGGGAGCCCAG

GCTTACGCGGCCGGGTTAGTAGTAAATGATAACGATCTGCGAAACGACCTTGCCTGGCTT

TCCGATCGCGGGGTTATCCATCTGAGCCTGTCGACCTGGCCGCTGAGCCAGGAAGAGATC

ACCCGGGCGTTAAAAAAAGCTAAACCGTCCTATTCTTCTGAGCAAGTGGTGCTGGCCCGT

ATCAATCAGCGACTGTCAGCGTTAAAAGCCGATTTTCGGGTCACCGGCTACGCCTCAACC

GACCAGCCGGGCACTCCGCAGGGGTTTGGTCAGACGCAGCCGGCAGATAACTCCTTAGGC

CTGGCGTTCAACAACAGCGGCGAGTGGTGGGATGTCCACCTCCAGGGCAACGTTGAGGGG

GGAGAGCGGATCAGCAACGGTTCGCGC

>P3_S1811_wzi186

ATGATAAAAATTGCGCGCATTGCCGTGACGTTGGGTTTGCTTTCCTCACTGGGAGCCCAG

GCTTACGCGGCTGGGTTAGTGGTAAATGACAACGACTTACGTAACGACCTGGCCTGGCTT

TCCGATCGCGGGGTCATCCATCTGAGCCTGTCGACCTGGCCGCTGAGCCAGGAAGAGATC

GCCCGGGCGCTAAAGAAGGCCAAGCCTTCCTATTCTTCTGAGCAAGTGGTGCTGGCTCGT

ATTAACCAGCGACTGTCTGCTTTAAAAGCCGATGTCAGGGTCACCGGCTACACTTCAACC

GACCAGCCGGGTACTCCGCAGGGGTTTGGCCAGACGCAGCCGGCAGATAACTCGTTAGGC

CTGGCATTCAACAACAGTGGCGAGTGGTGGGACGTACACCTCCAGGGTAACGTTGAAGGG

GGAGAGCGGATCAGCAACGGATCGCGC

>P3_S1812_wzi186

ATGATAAAAATTGCGCGCATTGCCGTGACGTTGGGTTTGCTTTCCTCACTGGGAGCCCAG

GCTTACGCGGCTGGGTTAGTGGTAAATGACAACGACTTACGTAACGACCTGGCCTGGCTT

TCCGATCGCGGGGTCATCCATCTGAGCCTGTCGACCTGGCCGCTGAGCCAGGAAGAGATC

GCCCGGGCGCTAAAGAAGGCCAAGCCTTCCTATTCTTCTGAGCAAGTGGTGCTGGCTCGT

ATTAACCAGCGACTGTCTGCTTTAAAAGCCGATGTCAGGGTCACCGGCTACACTTCAACC

GACCAGCCGGGTACTCCGCAGGGGTTTGGCCAGACGCAGCCGGCAGATAACTCGTTAGGC

CTGGCATTCAACAACAGTGGCGAGTGGTGGGACGTACACCTCCAGGGTAACGTTGAAGGG

GGAGAGCGGATCAGCAACGGATCGCGC

>P3_S1813_wzi186

ATGATAAAAATTGCGCGCATTGCCGTGACGTTGGGTTTGCTTTCCTCACTGGGAGCCCAG

GCTTACGCGGCTGGGTTAGTGGTAAATGACAACGACTTACGTAACGACCTGGCCTGGCTT

TCCGATCGCGGGGTCATCCATCTGAGCCTGTCGACCTGGCCGCTGAGCCAGGAAGAGATC

GCCCGGGCGCTAAAGAAGGCCAAGCCTTCCTATTCTTCTGAGCAAGTGGTGCTGGCTCGT

ATTAACCAGCGACTGTCTGCTTTAAAAGCCGATGTCAGGGTCACCGGCTACACTTCAACC

GACCAGCCGGGTACTCCGCAGGGGTTTGGCCAGACGCAGCCGGCAGATAACTCGTTAGGC

CTGGCATTCAACAACAGTGGCGAGTGGTGGGACGTACACCTCCAGGGTAACGTTGAAGGG

GGAGAGCGGATCAGCAACGGATCGCGC

>P4_B2008_wzi114

ATGATAAAAATTGCGCGCATTGCCGTGACGTTGGGTTTGCTTTCCTCACTGGGAGCCCAG

GCTTACGCGGCCGGGTTAGTGGTAAATGACAACGACTTACGTAACGACCTGGCCTGGCTT

TCCGATCGCGGGGTCATCCATCTGAGCCTGTCGACCTGGCCGCTGAGCCAGGAAGAGATT

GCTCGGGCGCTAAAGAAGGCCAAGCCGTCTTATTCTTCTGAGCAAGTAGTTCTGGCCCGT

ATCAACCAGCGACTGTCTGCCTTAAAAGCGGATTTCCGGGTCACCGGCTACACTTCAACC

GATCAGCCGGGCACACCGCAGGGGTTTGGCCAGACGCAGCCGGCGGATAACTCGTTAGGC

CTGGCGTTCAACAACAGCGGCGAGTGGTGGGATGTCCATCTCCAGGGCAATGTTGAGGGG

GGAGAGCGGATTAGCAACGGGTCGCGC

>P4_S1872_UK1

ATGATAAAAATTGCGCGCATTGCCGTGACGTTGGGTTTGCTTTCCTCCCTGGGAGCCCAG

GCTTACGCGGCCGGGTTAGTAGTAAATGACAACGACTTGCGTAACGACCTGGCCTGGCTT

TCCGATCGTGGGGTCATCCATCTGAGCCTGTCGACTTGGCCGCTGAGCCAGGAAGAGATC

TCCCGGGCGCTAAAAAAGGCCAAACCGTCCTATTCTTCTGAGCAAGTGGTGCTGGCTCGT

ATTAACCAGCGACTGTCTGCCTTAAAAGCGGATTTCCGGGTTACCGGCTACACTTCAACC

GATCAGCCGGGCACTCCGCAGGGGTTTGGTCAGACACAGCCGGCAGATAACTCATTAGGC

CTGGCGTTCAACAACAGCGGCGAGTGGTGGGATATCCACCTGCAGGGTAACGTCGAAGGA

GGGGAGCGGATCAGCAACGGATCGCGC

>P4_S1873_UK1

ATGATAAAAATTGCGCGCATTGCCGTGACGTTGGGTTTGCTTTCCTCCCTGGGAGCCCAG

GCTTACGCGGCCGGGTTAGTAGTAAATGACAACGACTTGCGTAACGACCTGGCCTGGCTT

TCCGATCGTGGGGTCATCCATCTGAGCCTGTCGACTTGGCCGCTGAGCCAGGAAGAGATC

TCCCGGGCGCTAAAAAAGGCCAAACCGTCCTATTCTTCTGAGCAAGTGGTGCTGGCTCGT

ATTAACCAGCGACTGTCTGCCTTAAAAGCGGATTTCCGGGTTACCGGCTACACTTCAACC

GATCAGCCGGGCACTCCGCAGGGGTTTGGTCAGACACAGCCGGCAGATAACTCATTAGGC

CTGGCGTTCAACAACAGCGGCGAGTGGTGGGATATCCACCTGCAGGGTAACGTCGAAGGA

GGGGAGCGGATCAGCAACGGATCGCGC

>P4_S1874_UK1

ATGATAAAAATTGCGCGCATTGCCGTGACGTTGGGTTTGCTTTCCTCCCTGGGAGCCCAG

GCTTACGCGGCCGGGTTAGTAGTAAATGACAACGACTTGCGTAACGACCTGGCCTGGCTT

TCCGATCGTGGGGTCATCCATCTGAGCCTGTCGACTTGGCCGCTGAGCCAGGAAGAGATC

TCCCGGGCGCTAAAAAAGGCCAAACCGTCCTATTCTTCTGAGCAAGTGGTGCTGGCTCGT

ATTAACCAGCGACTGTCTGCCTTAAAAGCGGATTTCCGGGTTACCGGCTACACTTCAACC

GATCAGCCGGGCACTCCGCAGGGGTTTGGTCAGACACAGCCGGCAGATAACTCATTAGGC

CTGGCGTTCAACAACAGCGGCGAGTGGTGGGATATCCACCTGCAGGGTAACGTCGAAGGA

GGGGAGCGGATCAGCAACGGATCGCGC

>P5_B2146_wzi57_K57

ATGATAAAAATTGCGCGCATTGCCGTGACGCTGGGCTTGCTTTCCTCACTGGGAGCCCAG

GCTTACGCGGCCGGGTTAGTAGTAAATGATAACGATCTGCGAAACGACCTTGCCTGGCTT

TCCGATCGCGGGGTCATCCATCTGAGCCTGTCGACCTGGCCGCTTAGCCAGGAAGAGATC

GCCCGGGCGCTAAAAAAAGCTAAACCGTCCTATTCTTCTGAGCAAGTAGTGCTGGCCCGT

ATCAACCAGCGACTGTCTGCTTTAAAAGCCGATTTCCGGGTCACCGGCTACACCTCAACC

GACCAGCCTGGCACCCCGCAGGGGTTTGGCCAGACGCAGCCGGCAGATAACTCGTTAGGC

CTGGCGTTTAACAACAGCGGCGAGTGGTGGGACGTTCACCTCCAGGGCAACGTTGAAGGG

GGAGAGCGAATCAGCAACGGATCGCGT

>P5_S2069_wzi57_K57

ATGATAAAAATTGCGCGCATTGCCGTGACGCTGGGCTTGCTTTCCTCACTGGGAGCCCAG

GCTTACGCGGCCGGGTTAGTAGTAAATGATAACGATCTGCGAAACGACCTTGCCTGGCTT

TCCGATCGCGGGGTCATCCATCTGAGCCTGTCGACCTGGCCGCTTAGCCAGGAAGAGATC

GCCCGGGCGCTAAAAAAAGCTAAACCGTCCTATTCTTCTGAGCAAGTAGTGCTGGCCCGT

ATCAACCAGCGACTGTCTGCTTTAAAAGCCGATTTCCGGGTCACCGGCTACACCTCAACC

GACCAGCCTGGCACCCCGCAGGGGTTTGGCCAGACGCAGCCGGCAGATAACTCGTTAGGC

CTGGCGTTTAACAACAGCGGCGAGTGGTGGGACGTTCACCTCCAGGGCAACGTTGAAGGG

GGAGAGCGAATCAGCAACGGATCGCGT

>P5_S2070_wzi57_K57

ATGATAAAAATTGCGCGCATTGCCGTGACGCTGGGCTTGCTTTCCTCACTGGGAGCCCAG

GCTTACGCGGCCGGGTTAGTAGTAAATGATAACGATCTGCGAAACGACCTTGCCTGGCTT

TCCGATCGCGGGGTCATCCATCTGAGCCTGTCGACCTGGCCGCTTAGCCAGGAAGAGATC

GCCCGGGCGCTAAAAAAAGCTAAACCGTCCTATTCTTCTGAGCAAGTAGTGCTGGCCCGT

ATCAACCAGCGACTGTCTGCTTTAAAAGCCGATTTCCGGGTCACCGGCTACACCTCAACC

GACCAGCCTGGCACCCCGCAGGGGTTTGGCCAGACGCAGCCGGCAGATAACTCGTTAGGC

CTGGCGTTTAACAACAGCGGCGAGTGGTGGGACGTTCACCTCCAGGGCAACGTTGAAGGG

GGAGAGCGAATCAGCAACGGATCGCGT

>P5_S2071_wzi57_K57

ATGATAAAAATTGCGCGCATTGCCGTGACGCTGGGCTTGCTTTCCTCACTGGGAGCCCAG

GCTTACGCGGCCGGGTTAGTAGTAAATGATAACGATCTGCGAAACGACCTTGCCTGGCTT

TCCGATCGCGGGGTCATCCATCTGAGCCTGTCGACCTGGCCGCTTAGCCAGGAAGAGATC

GCCCGGGCGCTAAAAAAAGCTAAACCGTCCTATTCTTCTGAGCAAGTAGTGCTGGCCCGT

ATCAACCAGCGACTGTCTGCTTTAAAAGCCGATTTCCGGGTCACCGGCTACACCTCAACC

GACCAGCCTGGCACCCCGCAGGGGTTTGGCCAGACGCAGCCGGCAGATAACTCGTTAGGC

CTGGCGTTTAACAACAGCGGCGAGTGGTGGGACGTTCACCTCCAGGGCAACGTTGAAGGG

GGAGAGCGAATCAGCAACGGATCGCGT

>P6_R728_wzi82_K23

ATGATAAAAATTGCGCGCATTGCCGTGACGTTGGGTTTGCTTTCCTCACTGGGAGCCCAG

GCTTACGCGGCCGGGTTAGTGGTAAATGACAACGACTTACGTAACGACCTGGCCTGGCTT

TCCGATCGCGGGGTCATCCATCTGAGCCTGTCGACCTGGCCGCTGAGCCAGGAAGAGATC

TCCCGGGCGCTAAAAAAGGCCAAACCGTCCTATTCTTCTGAACAAGTGGTGCTGGCACGT

ATTAACCAGCGACTGTCTGCCTTAAAAGCCGATTTCCGGGTCACCGGCTACACTTCAACC

GATCAGCCGGGCACTCCGCAGGGGTTTGGTCAGACACAGCCGGCAGATAACTCGTTAGGC

CTGGCGTTCAACAACAGCGGCGAGTGGTGGGATATCCACCTCCAGGGCAACGTTGAAGGG

GGAGAGCGGATCAGCAACGGATCGCGC

>P6_S997_wzi82_K23

ATGATAAAAATTGCGCGCATTGCCGTGACGTTGGGTTTGCTTTCCTCACTGGGAGCCCAG

GCTTACGCGGCCGGGTTAGTGGTAAATGACAACGACTTACGTAACGACCTGGCCTGGCTT

TCCGATCGCGGGGTCATCCATCTGAGCCTGTCGACCTGGCCGCTGAGCCAGGAAGAGATC

TCCCGGGCGCTAAAAAAGGCCAAACCGTCCTATTCTTCTGAACAAGTGGTGCTGGCACGT

ATTAACCAGCGACTGTCTGCCTTAAAAGCCGATTTCCGGGTCACCGGCTACACTTCAACC

GATCAGCCGGGCACTCCGCAGGGGTTTGGTCAGACACAGCCGGCAGATAACTCGTTAGGC

CTGGCGTTCAACAACAGCGGCGAGTGGTGGGATATCCACCTCCAGGGCAACGTTGAAGGG

GGAGAGCGGATCAGCAACGGATCGCGC

>P6_S998_wzi82_K23

ATGATAAAAATTGCGCGCATTGCCGTGACGTTGGGTTTGCTTTCCTCACTGGGAGCCCAG

GCTTACGCGGCCGGGTTAGTGGTAAATGACAACGACTTACGTAACGACCTGGCCTGGCTT

TCCGATCGCGGGGTCATCCATCTGAGCCTGTCGACCTGGCCGCTGAGCCAGGAAGAGATC

TCCCGGGCGCTAAAAAAGGCCAAACCGTCCTATTCTTCTGAACAAGTGGTGCTGGCACGT

ATTAACCAGCGACTGTCTGCCTTAAAAGCCGATTTCCGGGTCACCGGCTACACTTCAACC

GATCAGCCGGGCACTCCGCAGGGGTTTGGTCAGACACAGCCGGCAGATAACTCGTTAGGC

CTGGCGTTCAACAACAGCGGCGAGTGGTGGGATATCCACCTCCAGGGCAACGTTGAAGGG

GGAGAGCGGATCAGCAACGGATCGCGC

>P6_S999_wzi82_K23

ATGATAAAAATTGCGCGCATTGCCGTGACGTTGGGTTTGCTTTCCTCACTGGGAGCCCAG

GCTTACGCGGCCGGGTTAGTGGTAAATGACAACGACTTACGTAACGACCTGGCCTGGCTT

TCCGATCGCGGGGTCATCCATCTGAGCCTGTCGACCTGGCCGCTGAGCCAGGAAGAGATC

TCCCGGGCGCTAAAAAAGGCCAAACCGTCCTATTCTTCTGAACAAGTGGTGCTGGCACGT

ATTAACCAGCGACTGTCTGCCTTAAAAGCCGATTTCCGGGTCACCGGCTACACTTCAACC

GATCAGCCGGGCACTCCGCAGGGGTTTGGTCAGACACAGCCGGCAGATAACTCGTTAGGC

CTGGCGTTCAACAACAGCGGCGAGTGGTGGGATATCCACCTCCAGGGCAACGTTGAAGGG

GGAGAGCGGATCAGCAACGGATCGCGC

>P7_R735_wzi101_K24

ATGATAAAAATTGCGCGCATTGCCGTGACGTTGGGTTTGCTTTCCTCCCTGGGAGCCCAG

GCTTACGCGGCCGGGTTAGTGGTAAATGACAACGACTTGCGTAACGACCTGGCCTGGCTT

TCCGATCGCGGGGTCATCCATCTGAGCCTGTCGACCTGGCCGCTGAGCCAGGAAGAGATC

GCCCGGGCACTGAAAAAGGCTAAACCGTCCTATTCTTCTGAACAAGTGGTGCTGGCGCGT

ATTAACCAGCGACTGTCTGCCTTGAAAGCCGATTTTCGGGTCACCGGCTACACTTCAACC

GACAAGCCGGGCACTCCGCAGGGGTTTGGCCAGACGCAGCCGGCAGATAATTCGTTAGGC

CTGGCGTTCAATAACAGCGGTGAGTGGTGGGATGTTCACCTGCAGGGCAACGTTGAAGGG

GGAGAGCGGATCAGCAACGGATCGCGC

>P7_S1003_wzi101_K24

ATGATAAAAATTGCGCGCATTGCCGTGACGTTGGGTTTGCTTTCCTCCCTGGGAGCCCAG

GCTTACGCGGCCGGGTTAGTGGTAAATGACAACGACTTGCGTAACGACCTGGCCTGGCTT

TCCGATCGCGGGGTCATCCATCTGAGCCTGTCGACCTGGCCGCTGAGCCAGGAAGAGATC

GCCCGGGCACTGAAAAAGGCTAAACCGTCCTATTCTTCTGAACAAGTGGTGCTGGCGCGT

ATTAACCAGCGACTGTCTGCCTTGAAAGCCGATTTTCGGGTCACCGGCTACACTTCAACC

GACAAGCCGGGCACTCCGCAGGGGTTTGGCCAGACGCAGCCGGCAGATAATTCGTTAGGC

CTGGCGTTCAATAACAGCGGTGAGTGGTGGGATGTTCACCTGCAGGGCAACGTTGAAGGG

GGAGAGCGGATCAGCAACGGATCGCGC

>P7_S1004_wzi101_K24

ATGATAAAAATTGCGCGCATTGCCGTGACGTTGGGTTTGCTTTCCTCCCTGGGAGCCCAG

GCTTACGCGGCCGGGTTAGTGGTAAATGACAACGACTTGCGTAACGACCTGGCCTGGCTT

TCCGATCGCGGGGTCATCCATCTGAGCCTGTCGACCTGGCCGCTGAGCCAGGAAGAGATC

GCCCGGGCACTGAAAAAGGCTAAACCGTCCTATTCTTCTGAACAAGTGGTGCTGGCGCGT

ATTAACCAGCGACTGTCTGCCTTGAAAGCCGATTTTCGGGTCACCGGCTACACTTCAACC

GACAAGCCGGGCACTCCGCAGGGGTTTGGCCAGACGCAGCCGGCAGATAATTCGTTAGGC

CTGGCGTTCAATAACAGCGGTGAGTGGTGGGATGTTCACCTGCAGGGCAACGTTGAAGGG

GGAGAGCGGATCAGCAACGGATCGCGC

>P7_S1005_wzi101_K24

ATGATAAAAATTGCGCGCATTGCCGTGACGTTGGGTTTGCTTTCCTCCCTGGGAGCCCAG

GCTTACGCGGCCGGGTTAGTGGTAAATGACAACGACTTGCGTAACGACCTGGCCTGGCTT

TCCGATCGCGGGGTCATCCATCTGAGCCTGTCGACCTGGCCGCTGAGCCAGGAAGAGATC

GCCCGGGCACTGAAAAAGGCTAAACCGTCCTATTCTTCTGAACAAGTGGTGCTGGCGCGT

ATTAACCAGCGACTGTCTGCCTTGAAAGCCGATTTTCGGGTCACCGGCTACACTTCAACC

GACAAGCCGGGCACTCCGCAGGGGTTTGGCCAGACGCAGCCGGCAGATAATTCGTTAGGC

CTGGCGTTCAATAACAGCGGTGAGTGGTGGGATGTTCACCTGCAGGGCAACGTTGAAGGG

GGAGAGCGGATCAGCAACGGATCGCGC

>P8_R733_wzi96_K38

ATGATAAAAATTGCGCGCATTGCCGTGACGTTGGGTTTGCTTTCCTCACTGGGAGCCCAG

GCTTACGCGGCCGGGTTAGTGGTAAATGACAACGACTTGCGTAACGACCTGGCCTGGCTT

TCCGATCGCGGGGTCATCCATCTGAGCCTGTCGACGTGGCCGCTGAGCCAGGAAGAGATC

GCCCGGGCGCTGAAAAAGGCCAAACCTTCCTATTCTTCTGAGCAAGTGGTGCTGGCGCGT

ATTAACCAGCGACTGTCTGCCTTAAAAGCGGATTTCCGGGTCACCGGCTACACTTCAACC

GATCAGCCAGGCACTCCGCAGGGGTTTGGTCAGACGCAGTCGGCGGATAATTCGTTAGGC

CTGGCGTTCAACAACAGCGGCGAGTGGTGGGACGTCCACCTTCAGGGCAATGTTGAAGGG

GGAGAGCGGATCAGCAACGGATCGCGC

>P8_R734_wzi96_K38

ATGATAAAAATTGCGCGCATTGCCGTGACGTTGGGTTTGCTTTCCTCACTGGGAGCCCAG

GCTTACGCGGCCGGGTTAGTGGTAAATGACAACGACTTGCGTAACGACCTGGCCTGGCTT

TCCGATCGCGGGGTCATCCATCTGAGCCTGTCGACGTGGCCGCTGAGCCAGGAAGAGATC

GCCCGGGCGCTGAAAAAGGCCAAACCTTCCTATTCTTCTGAGCAAGTGGTGCTGGCGCGT

ATTAACCAGCGACTGTCTGCCTTAAAAGCGGATTTCCGGGTCACCGGCTACACTTCAACC

GATCAGCCAGGCACTCCGCAGGGGTTTGGTCAGACGCAGTCGGCGGATAATTCGTTAGGC

CTGGCGTTCAACAACAGCGGCGAGTGGTGGGACGTCCACCTTCAGGGCAATGTTGAAGGG

GGAGAGCGGATCAGCAACGGATCGCGC

>P8_S1043_wzi96_K38

ATGATAAAAATTGCGCGCATTGCCGTGACGTTGGGTTTGCTTTCCTCACTGGGAGCCCAG

GCTTACGCGGCCGGGTTAGTGGTAAATGACAACGACTTGCGTAACGACCTGGCCTGGCTT

TCCGATCGCGGGGTCATCCATCTGAGCCTGTCGACGTGGCCGCTGAGCCAGGAAGAGATC

GCCCGGGCGCTGAAAAAGGCCAAACCTTCCTATTCTTCTGAGCAAGTGGTGCTGGCGCGT

ATTAACCAGCGACTGTCTGCCTTAAAAGCGGATTTCCGGGTCACCGGCTACACTTCAACC

GATCAGCCAGGCACTCCGCAGGGGTTTGGTCAGACGCAGTCGGCGGATAATTCGTTAGGC

CTGGCGTTCAACAACAGCGGCGAGTGGTGGGACGTCCACCTTCAGGGCAATGTTGAAGGG

GGAGAGCGGATCAGCAACGGATCGCGC

>P8_S1044_wzi96_K38

ATGATAAAAATTGCGCGCATTGCCGTGACGTTGGGTTTGCTTTCCTCACTGGGAGCCCAG

GCTTACGCGGCCGGGTTAGTGGTAAATGACAACGACTTGCGTAACGACCTGGCCTGGCTT

TCCGATCGCGGGGTCATCCATCTGAGCCTGTCGACGTGGCCGCTGAGCCAGGAAGAGATC

GCCCGGGCGCTGAAAAAGGCCAAACCTTCCTATTCTTCTGAGCAAGTGGTGCTGGCGCGT

ATTAACCAGCGACTGTCTGCCTTAAAAGCGGATTTCCGGGTCACCGGCTACACTTCAACC

GATCAGCCAGGCACTCCGCAGGGGTTTGGTCAGACGCAGTCGGCGGATAATTCGTTAGGC

CTGGCGTTCAACAACAGCGGCGAGTGGTGGGACGTCCACCTTCAGGGCAATGTTGAAGGG

GGAGAGCGGATCAGCAACGGATCGCGC

>P8_S1045_wzi453

ATGATAAAAATTGCGCGCATTGCCGTGACGCTGGGTTTGCTTTCCTCACTGGGAGCCCAG

GCTTACGCGGCCGGGTTAGTAGTAAATGATAACGATCTGCGAAACGACCTTGCCTGGCTT

TCCGATCGCGGGGTCATCCATCTGAGCCTGTCGACCTGGCCGCTAAGTCAGGAAGAGATC

GCCCGGGCGCTAAAAAAGGCCAAACCTTCCTATTCTTCTGAGCAAGTGGTTCTGGCTCGT

ATCAATCAGCGACTGTCAGCGCTAAAAGCAGATTTCCGGGTCACCGGCTACACCTCAACT

GACCAACCGGGCACTCCGCAGGGGTTTGGTCAGACGCAGCCGGCAGATAACTCGTTAGGC

CTGGCGTTTAACAATAGCGGCGAATGGTGGGATGTCCACCTTCAGGGTAACGTCGAAGGG

GGGGAGCGGATCAGTAACGGATCGCGC

>P9_R1317_wzi90_K37

ATGATAAAAATTGCGCGCATTGCCGTGACGTTGGGTTTGCTTTCCTCCCTGGGAGCCCAG

GCTTACGCGGCCGGGTTAGTGGTAAATGACAACGACTTACGTAACGACCTGGCCTGGCTT

TCCGATCGCGGGGTCATCCATCTGAGCCTGTCGACCTGGCCGCTGAGCCAGGAAGAGATT

GCTCGGGCGCTAAAGAAGGCCAAGCCGTCTTATTCTTCTGAGCAAGTAGTTCTGGCCCGT

ATCAACCAGCGACTGTCTGCCTTAAAAGCGGATTTCCGGGTCACCGGCTACACTTCAACC

GATCAGCCGGGCACACCGCAGGGGTTTGGTCAGACACAGCCGGCAGATAACTCGTTAGGC

CTGGCGTTCAACAACAGCGGCGAGTGGTGGGATGTCCATCTCCAGGGCAATGTTGAGGGG

GGAGAGCGGATTAGCAACGGGTCGCGC

>P9_S1223_wzi90_K37

ATGATAAAAATTGCGCGCATTGCCGTGACGTTGGGTTTGCTTTCCTCCCTGGGAGCCCAG

GCTTACGCGGCCGGGTTAGTGGTAAATGACAACGACTTACGTAACGACCTGGCCTGGCTT

TCCGATCGCGGGGTCATCCATCTGAGCCTGTCGACCTGGCCGCTGAGCCAGGAAGAGATT

GCTCGGGCGCTAAAGAAGGCCAAGCCGTCTTATTCTTCTGAGCAAGTAGTTCTGGCCCGT

ATCAACCAGCGACTGTCTGCCTTAAAAGCGGATTTCCGGGTCACCGGCTACACTTCAACC

GATCAGCCGGGCACACCGCAGGGGTTTGGTCAGACACAGCCGGCAGATAACTCGTTAGGC

CTGGCGTTCAACAACAGCGGCGAGTGGTGGGATGTCCATCTCCAGGGCAATGTTGAGGGG

GGAGAGCGGATTAGCAACGGGTCGCGC

>P9_S1224_wzi90_K37

ATGATAAAAATTGCGCGCATTGCCGTGACGTTGGGTTTGCTTTCCTCCCTGGGAGCCCAG

GCTTACGCGGCCGGGTTAGTGGTAAATGACAACGACTTACGTAACGACCTGGCCTGGCTT

TCCGATCGCGGGGTCATCCATCTGAGCCTGTCGACCTGGCCGCTGAGCCAGGAAGAGATT

GCTCGGGCGCTAAAGAAGGCCAAGCCGTCTTATTCTTCTGAGCAAGTAGTTCTGGCCCGT

ATCAACCAGCGACTGTCTGCCTTAAAAGCGGATTTCCGGGTCACCGGCTACACTTCAACC

GATCAGCCGGGCACACCGCAGGGGTTTGGTCAGACACAGCCGGCAGATAACTCGTTAGGC

CTGGCGTTCAACAACAGCGGCGAGTGGTGGGATGTCCATCTCCAGGGCAATGTTGAGGGG

GGAGAGCGGATTAGCAACGGGTCGCGC

>P9_S1225_wzi90_K37

ATGATAAAAATTGCGCGCATTGCCGTGACGTTGGGTTTGCTTTCCTCCCTGGGAGCCCAG

GCTTACGCGGCCGGGTTAGTGGTAAATGACAACGACTTACGTAACGACCTGGCCTGGCTT

TCCGATCGCGGGGTCATCCATCTGAGCCTGTCGACCTGGCCGCTGAGCCAGGAAGAGATT

GCTCGGGCGCTAAAGAAGGCCAAGCCGTCTTATTCTTCTGAGCAAGTAGTTCTGGCCCGT

ATCAACCAGCGACTGTCTGCCTTAAAAGCGGATTTCCGGGTCACCGGCTACACTTCAACC

GATCAGCCGGGCACACCGCAGGGGTTTGGTCAGACACAGCCGGCAGATAACTCGTTAGGC

CTGGCGTTCAACAACAGCGGCGAGTGGTGGGATGTCCATCTCCAGGGCAATGTTGAGGGG

GGAGAGCGGATTAGCAACGGGTCGCGC

>P10_R1637_wzi372

ATGATAAAAATTGCGCGCATTGCCGTGACGTTGGGTTTGCTTTCCTCCCTGGGAGCCCAG

GCTTACGCGGCCGGGTTAGTGGTAAATGACAACGACTTGCGTAACGACCTGGCCTGGCTT

TCCGATCGCGGGGTCATCCATCTGAGCCTGTCGACCTGGCCGCTGAGCCAGGAAGAGATC

GCCCGGGCACTGAAAAAGGCTAAACCGTCCTATTCTTCTGAACAAGTGGTGCTGGCGCGT

ATTAACCAGCGACTGTCTGCCTTAAAAGCGGATTTCCGGGTCACCGGCTACACTTCAACC

GATCAGCCGGGCACTCCGCAGGGGTTTGGTCAGACACAGCCGGCAGATAACTCGTTAGGC

CTGGCGTTCAACAACAGCGGCGAGTGGTGGGATGTCCACCTCCAGGGCAACGTCGAAGGA

GGGGAGCGGATCAGCAACGGCTCGCGC

>P10_S1584_wzi372

ATGATAAAAATTGCGCGCATTGCCGTGACGTTGGGTTTGCTTTCCTCCCTGGGAGCCCAG

GCTTACGCGGCCGGGTTAGTGGTAAATGACAACGACTTGCGTAACGACCTGGCCTGGCTT

TCCGATCGCGGGGTCATCCATCTGAGCCTGTCGACCTGGCCGCTGAGCCAGGAAGAGATC

GCCCGGGCACTGAAAAAGGCTAAACCGTCCTATTCTTCTGAACAAGTGGTGCTGGCGCGT

ATTAACCAGCGACTGTCTGCCTTAAAAGCGGATTTCCGGGTCACCGGCTACACTTCAACC

GATCAGCCGGGCACTCCGCAGGGGTTTGGTCAGACACAGCCGGCAGATAACTCGTTAGGC

CTGGCGTTCAACAACAGCGGCGAGTGGTGGGATGTCCACCTCCAGGGCAACGTCGAAGGA

GGGGAGCGGATCAGCAACGGCTCGCGC

>P10_S1585_wzi372

ATGATAAAAATTGCGCGCATTGCCGTGACGTTGGGTTTGCTTTCCTCCCTGGGAGCCCAG

GCTTACGCGGCCGGGTTAGTGGTAAATGACAACGACTTGCGTAACGACCTGGCCTGGCTT

TCCGATCGCGGGGTCATCCATCTGAGCCTGTCGACCTGGCCGCTGAGCCAGGAAGAGATC

GCCCGGGCACTGAAAAAGGCTAAACCGTCCTATTCTTCTGAACAAGTGGTGCTGGCGCGT

ATTAACCAGCGACTGTCTGCCTTAAAAGCGGATTTCCGGGTCACCGGCTACACTTCAACC

GATCAGCCGGGCACTCCGCAGGGGTTTGGTCAGACACAGCCGGCAGATAACTCGTTAGGC

CTGGCGTTCAACAACAGCGGCGAGTGGTGGGATGTCCACCTCCAGGGCAACGTCGAAGGA

GGGGAGCGGATCAGCAACGGCTCGCGC

>P11_R1950_wzi150

ATGATAAAAATTGCGCGCATTGCCGTGACGTTGGGTTTGCTTTCCTCACTGGGAGCCCAG

GCTTTCGCGGCCGGGTTAGTGGTAAATGACAACGACTTGCGTAACGACCTGGCCTGGCTT

TCCGATCGCGGGGTCATCCATCTGAGCCTGTCGACTTGGCCGCTGAGCCAGGAAGAAATC

TCCCGGGCGCTAAAAAAGGCCAAACCGTCCTATTCTTCTGAGCAAGTGGTGCTGGCTCGT

ATTAACCAGCGACTGTCTGCCTTAAAAGCCGATTTTCGGGTCACTGGTTACACCTCAACC

GATCAGCCGGGCACTCCGCAGGGGTTTGGTCAGACACAGCCGGCAGATAACTCGTTAGGC

CTGGCGTTCAACAACAGCGGCGAGTGGTGGGATATCCACCTCCAGGGCAACGTCGAAGGA

GGGGAGCGGATCAGCAACGGATCGCGC

>P11_S1875_wzi150

ATGATAAAAATTGCGCGCATTGCCGTGACGTTGGGTTTGCTTTCCTCACTGGGAGCCCAG

GCTTTCGCGGCCGGGTTAGTGGTAAATGACAACGACTTGCGTAACGACCTGGCCTGGCTT

TCCGATCGCGGGGTCATCCATCTGAGCCTGTCGACTTGGCCGCTGAGCCAGGAAGAAATC

TCCCGGGCGCTAAAAAAGGCCAAACCGTCCTATTCTTCTGAGCAAGTGGTGCTGGCTCGT

ATTAACCAGCGACTGTCTGCCTTAAAAGCCGATTTTCGGGTCACTGGTTACACCTCAACC

GATCAGCCGGGCACTCCGCAGGGGTTTGGTCAGACACAGCCGGCAGATAACTCGTTAGGC

CTGGCGTTCAACAACAGCGGCGAGTGGTGGGATATCCACCTCCAGGGCAACGTCGAAGGA

GGGGAGCGGATCAGCAACGGATCGCGC

>P11_S1876_wzi150

ATGATAAAAATTGCGCGCATTGCCGTGACGTTGGGTTTGCTTTCCTCACTGGGAGCCCAG

GCTTTCGCGGCCGGGTTAGTGGTAAATGACAACGACTTGCGTAACGACCTGGCCTGGCTT

TCCGATCGCGGGGTCATCCATCTGAGCCTGTCGACTTGGCCGCTGAGCCAGGAAGAAATC

TCCCGGGCGCTAAAAAAGGCCAAACCGTCCTATTCTTCTGAGCAAGTGGTGCTGGCTCGT

ATTAACCAGCGACTGTCTGCCTTAAAAGCCGATTTTCGGGTCACTGGTTACACCTCAACC

GATCAGCCGGGCACTCCGCAGGGGTTTGGTCAGACACAGCCGGCAGATAACTCGTTAGGC

CTGGCGTTCAACAACAGCGGCGAGTGGTGGGATATCCACCTCCAGGGCAACGTCGAAGGA

GGGGAGCGGATCAGCAACGGATCGCGC

>P12_R2005_wzi173

ATGATAAAAATTGCGCGCATTGCCGTGACGCTGGGTTTGCTTTCCTCCCTGGGAGCCCAG

GCTTACGCGGCCGGGTTAGTGGTAAATGACAACGACTTGCGTAACGACCTTGCCTGGCTT

TCCGATCGCGGGGTCATTCATCTGAGCCTGTCGACGTGGCCGCTGAGCCAGGAAGAGATA

GCCCGGGCGCTAAAAAAGGCCAAACCGTCTTATTCTTCTGAGCAAGTAGTTCTGGCCCGT

ATCAACCAGAGACTATCTGCCTTAAAAGCGGATTTCCGGGTCACCGGCTACACTTCAACC

GATCAGCCAGGCACTCCGCAGGGGTTTGGCCAGACGCAGCCGGCAGATAACTCGTTAGGC

CTGGCGTTCAACAACAGCGGCGAGTGGTGGGATATCCACCTCCAGGGCAACGTCGAAGGA

GGGGAGCGGATCAGCAACGGATCGCGC

>P12_S1967_wzi173

ATGATAAAAATTGCGCGCATTGCCGTGACGCTGGGTTTGCTTTCCTCCCTGGGAGCCCAG

GCTTACGCGGCCGGGTTAGTGGTAAATGACAACGACTTGCGTAACGACCTTGCCTGGCTT

TCCGATCGCGGGGTCATTCATCTGAGCCTGTCGACGTGGCCGCTGAGCCAGGAAGAGATA

GCCCGGGCGCTAAAAAAGGCCAAACCGTCTTATTCTTCTGAGCAAGTAGTTCTGGCCCGT

ATCAACCAGAGACTATCTGCCTTAAAAGCGGATTTCCGGGTCACCGGCTACACTTCAACC

GATCAGCCAGGCACTCCGCAGGGGTTTGGCCAGACGCAGCCGGCAGATAACTCGTTAGGC

CTGGCGTTCAACAACAGCGGCGAGTGGTGGGATATCCACCTCCAGGGCAACGTCGAAGGA

GGGGAGCGGATCAGCAACGGATCGCGC

>P12_S1968_wzi186

ATGATAAAAATTGCGCGCATTGCCGTGACGTTGGGTTTGCTTTCCTCACTGGGAGCCCAG

GCTTACGCGGCTGGGTTAGTGGTAAATGACAACGACTTACGTAACGACCTGGCCTGGCTT

TCCGATCGCGGGGTCATCCATCTGAGCCTGTCGACCTGGCCGCTGAGCCAGGAAGAGATC

GCCCGGGCGCTAAAGAAGGCCAAGCCTTCCTATTCTTCTGAGCAAGTGGTGCTGGCTCGT

ATTAACCAGCGACTGTCTGCTTTAAAAGCCGATGTCAGGGTCACCGGCTACACTTCAACC

GACCAGCCGGGTACTCCGCAGGGGTTTGGCCAGACGCAGCCGGCAGATAACTCGTTAGGC

CTGGCATTCAACAACAGTGGCGAGTGGTGGGACGTACACCTCCAGGGTAACGTTGAAGGG

GGAGAGCGGATCAGCAACGGATCGCGC

>P13_U616_wzi454

ATGATAAAAATTGCGCGCATTGCCGTGACGCTGGGCTTGCTTTCCTCACTGGGAGCCCAG

GCTTACGCGGCCGGGTTAGTAGTAAATGATAATGATCTGCGTAACGACCTTGCCTGGCTT

TCCGATCGCGGGGTCATCCATCTGAGCCTGTCGACCTGGCCGCTAAGTCAGGAAGAGATC

GCCCGGGCGCTAAAAAAGGCCAAACCTTCCTATTCTTCTGAGCAAGTGGTTCTGGCTCGT

ATCAATCAGCGACTGTCAGCGCTAAAAGCAGATTTCCGGGTCACCGGCTACACCTCAACC

GACCAACCGGGCACTCCGCAGGGGTTTGGTCAGACGCAGCCGGCGGATAACTCCTTAGGC

CTGGCGTTTAACAACAGCGGCGAATGGTGGGATGTCCACCTTCAGGGTAACGTCGATGGG

GGGGAGCGGATCAGTAACGGATCGCGC

>P13_S901_wzi454

ATGATAAAAATTGCGCGCATTGCCGTGACGCTGGGCTTGCTTTCCTCACTGGGAGCCCAG

GCTTACGCGGCCGGGTTAGTAGTAAATGATAATGATCTGCGTAACGACCTTGCCTGGCTT

TCCGATCGCGGGGTCATCCATCTGAGCCTGTCGACCTGGCCGCTAAGTCAGGAAGAGATC

GCCCGGGCGCTAAAAAAGGCCAAACCTTCCTATTCTTCTGAGCAAGTGGTTCTGGCTCGT

ATCAATCAGCGACTGTCAGCGCTAAAAGCAGATTTCCGGGTCACCGGCTACACCTCAACC

GACCAACCGGGCACTCCGCAGGGGTTTGGTCAGACGCAGCCGGCGGATAACTCCTTAGGC

CTGGCGTTTAACAACAGCGGCGAATGGTGGGATGTCCACCTTCAGGGTAACGTCGATGGG

GGGGAGCGGATCAGTAACGGATCGCGC

>P13_S902_wzi454

ATGATAAAAATTGCGCGCATTGCCGTGACGCTGGGCTTGCTTTCCTCACTGGGAGCCCAG

GCTTACGCGGCCGGGTTAGTAGTAAATGATAATGATCTGCGTAACGACCTTGCCTGGCTT

TCCGATCGCGGGGTCATCCATCTGAGCCTGTCGACCTGGCCGCTAAGTCAGGAAGAGATC

GCCCGGGCGCTAAAAAAGGCCAAACCTTCCTATTCTTCTGAGCAAGTGGTTCTGGCTCGT

ATCAATCAGCGACTGTCAGCGCTAAAAGCAGATTTCCGGGTCACCGGCTACACCTCAACC

GACCAACCGGGCACTCCGCAGGGGTTTGGTCAGACGCAGCCGGCGGATAACTCCTTAGGC

CTGGCGTTTAACAACAGCGGCGAATGGTGGGATGTCCACCTTCAGGGTAACGTCGATGGG

GGGGAGCGGATCAGTAACGGATCGCGC

>P13_S903_wzi454

ATGATAAAAATTGCGCGCATTGCCGTGACGCTGGGCTTGCTTTCCTCACTGGGAGCCCAG

GCTTACGCGGCCGGGTTAGTAGTAAATGATAATGATCTGCGTAACGACCTTGCCTGGCTT

TCCGATCGCGGGGTCATCCATCTGAGCCTGTCGACCTGGCCGCTAAGTCAGGAAGAGATC

GCCCGGGCGCTAAAAAAGGCCAAACCTTCCTATTCTTCTGAGCAAGTGGTTCTGGCTCGT

ATCAATCAGCGACTGTCAGCGCTAAAAGCAGATTTCCGGGTCACCGGCTACACCTCAACC

GACCAACCGGGCACTCCGCAGGGGTTTGGTCAGACGCAGCCGGCGGATAACTCCTTAGGC

CTGGCGTTTAACAACAGCGGCGAATGGTGGGATGTCCACCTTCAGGGTAACGTCGATGGG

GGGGAGCGGATCAGTAACGGATCGCGC

>P14_U714_wzi82_K23

ATGATAAAAATTGCGCGCATTGCCGTGACGTTGGGTTTGCTTTCCTCACTGGGAGCCCAG

GCTTACGCGGCCGGGTTAGTGGTAAATGACAACGACTTACGTAACGACCTGGCCTGGCTT

TCCGATCGCGGGGTCATCCATCTGAGCCTGTCGACCTGGCCGCTGAGCCAGGAAGAGATC

TCCCGGGCGCTAAAAAAGGCCAAACCGTCCTATTCTTCTGAACAAGTGGTGCTGGCACGT

ATTAACCAGCGACTGTCTGCCTTAAAAGCCGATTTCCGGGTCACCGGCTACACTTCAACC

GATCAGCCGGGCACTCCGCAGGGGTTTGGTCAGACACAGCCGGCAGATAACTCGTTAGGC

CTGGCGTTCAACAACAGCGGCGAGTGGTGGGATATCCACCTCCAGGGCAACGTTGAAGGG

GGAGAGCGGATCAGCAACGGATCGCGC

>P14_S688_wzi82_K23

ATGATAAAAATTGCGCGCATTGCCGTGACGTTGGGTTTGCTTTCCTCACTGGGAGCCCAG

GCTTACGCGGCCGGGTTAGTGGTAAATGACAACGACTTACGTAACGACCTGGCCTGGCTT

TCCGATCGCGGGGTCATCCATCTGAGCCTGTCGACCTGGCCGCTGAGCCAGGAAGAGATC

TCCCGGGCGCTAAAAAAGGCCAAACCGTCCTATTCTTCTGAACAAGTGGTGCTGGCACGT

ATTAACCAGCGACTGTCTGCCTTAAAAGCCGATTTCCGGGTCACCGGCTACACTTCAACC

GATCAGCCGGGCACTCCGCAGGGGTTTGGTCAGACACAGCCGGCAGATAACTCGTTAGGC

CTGGCGTTCAACAACAGCGGCGAGTGGTGGGATATCCACCTCCAGGGCAACGTTGAAGGG

GGAGAGCGGATCAGCAACGGATCGCGC

>P14_S689_wzi82_K23

ATGATAAAAATTGCGCGCATTGCCGTGACGTTGGGTTTGCTTTCCTCACTGGGAGCCCAG

GCTTACGCGGCCGGGTTAGTGGTAAATGACAACGACTTACGTAACGACCTGGCCTGGCTT

TCCGATCGCGGGGTCATCCATCTGAGCCTGTCGACCTGGCCGCTGAGCCAGGAAGAGATC

TCCCGGGCGCTAAAAAAGGCCAAACCGTCCTATTCTTCTGAACAAGTGGTGCTGGCACGT

ATTAACCAGCGACTGTCTGCCTTAAAAGCCGATTTCCGGGTCACCGGCTACACTTCAACC

GATCAGCCGGGCACTCCGCAGGGGTTTGGTCAGACACAGCCGGCAGATAACTCGTTAGGC

CTGGCGTTCAACAACAGCGGCGAGTGGTGGGATATCCACCTCCAGGGCAACGTTGAAGGG

GGAGAGCGGATCAGCAACGGATCGCGC

>P14_S690_wzi82_K23

ATGATAAAAATTGCGCGCATTGCCGTGACGTTGGGTTTGCTTTCCTCACTGGGAGCCCAG

GCTTACGCGGCCGGGTTAGTGGTAAATGACAACGACTTACGTAACGACCTGGCCTGGCTT

TCCGATCGCGGGGTCATCCATCTGAGCCTGTCGACCTGGCCGCTGAGCCAGGAAGAGATC

TCCCGGGCGCTAAAAAAGGCCAAACCGTCCTATTCTTCTGAACAAGTGGTGCTGGCACGT

ATTAACCAGCGACTGTCTGCCTTAAAAGCCGATTTCCGGGTCACCGGCTACACTTCAACC

GATCAGCCGGGCACTCCGCAGGGGTTTGGTCAGACACAGCCGGCAGATAACTCGTTAGGC

CTGGCGTTCAACAACAGCGGCGAGTGGTGGGATATCCACCTCCAGGGCAACGTTGAAGGG

GGAGAGCGGATCAGCAACGGATCGCGC

>P15_U664_wzi108_K80

ATGATAAAAATTGCGCGCATTGCCGTGACGTTGGGTTTGCTTTCCTCCCTGGGAGCCCAG

GCTTACGCAGCCGGGTTAGTGGTAAATGACAACGACTTGCGTAACGACCTGGCTTGGCTT

TCCGATCGCGGGGTCATCCATCTGAGCCTGTCGACCTGGCCGCTGAGCCAGGAAGAGATT

GCCCGGGCGCTGAAAAAGGCCAAACCGTCCTATTCTTCTGAGCAGGTAGTTCTGGCCCGT

ATCAACCAGAGACTGTCTGCCTTAAAAGCGGATTTCCGGGTCACCGGCTACACTTCAACA

GATCAGCCGGGCACTCCGCAGGGGTTTGGCCAGACGCAGCCGGCGGATAACTCGTTAGGC

CTGGCGTTCAACAACAGCGGCGAGTGGTGGGACGTCCACCTTCAGGGCAATGTTGAAGGG

GGAGAGCGGATCAGCAACGGATCGCGC

>P15_S767_wzi108_K80

ATGATAAAAATTGCGCGCATTGCCGTGACGTTGGGTTTGCTTTCCTCCCTGGGAGCCCAG

GCTTACGCAGCCGGGTTAGTGGTAAATGACAACGACTTGCGTAACGACCTGGCTTGGCTT

TCCGATCGCGGGGTCATCCATCTGAGCCTGTCGACCTGGCCGCTGAGCCAGGAAGAGATT

GCCCGGGCGCTGAAAAAGGCCAAACCGTCCTATTCTTCTGAGCAGGTAGTTCTGGCCCGT

ATCAACCAGAGACTGTCTGCCTTAAAAGCGGATTTCCGGGTCACCGGCTACACTTCAACA

GATCAGCCGGGCACTCCGCAGGGGTTTGGCCAGACGCAGCCGGCGGATAACTCGTTAGGC

CTGGCGTTCAACAACAGCGGCGAGTGGTGGGACGTCCACCTTCAGGGCAATGTTGAAGGG

GGAGAGCGGATCAGCAACGGATCGCGC

>P15_S768_wzi108_K80

ATGATAAAAATTGCGCGCATTGCCGTGACGTTGGGTTTGCTTTCCTCCCTGGGAGCCCAG

GCTTACGCAGCCGGGTTAGTGGTAAATGACAACGACTTGCGTAACGACCTGGCTTGGCTT

TCCGATCGCGGGGTCATCCATCTGAGCCTGTCGACCTGGCCGCTGAGCCAGGAAGAGATT

GCCCGGGCGCTGAAAAAGGCCAAACCGTCCTATTCTTCTGAGCAGGTAGTTCTGGCCCGT

ATCAACCAGAGACTGTCTGCCTTAAAAGCGGATTTCCGGGTCACCGGCTACACTTCAACA

GATCAGCCGGGCACTCCGCAGGGGTTTGGCCAGACGCAGCCGGCGGATAACTCGTTAGGC

CTGGCGTTCAACAACAGCGGCGAGTGGTGGGACGTCCACCTTCAGGGCAATGTTGAAGGG

GGAGAGCGGATCAGCAACGGATCGCGC

>P15_S769_wzi108_K80

ATGATAAAAATTGCGCGCATTGCCGTGACGTTGGGTTTGCTTTCCTCCCTGGGAGCCCAG

GCTTACGCAGCCGGGTTAGTGGTAAATGACAACGACTTGCGTAACGACCTGGCTTGGCTT

TCCGATCGCGGGGTCATCCATCTGAGCCTGTCGACCTGGCCGCTGAGCCAGGAAGAGATT

GCCCGGGCGCTGAAAAAGGCCAAACCGTCCTATTCTTCTGAGCAGGTAGTTCTGGCCCGT

ATCAACCAGAGACTGTCTGCCTTAAAAGCGGATTTCCGGGTCACCGGCTACACTTCAACA

GATCAGCCGGGCACTCCGCAGGGGTTTGGCCAGACGCAGCCGGCGGATAACTCGTTAGGC

CTGGCGTTCAACAACAGCGGCGAGTGGTGGGACGTCCACCTTCAGGGCAATGTTGAAGGG

GGAGAGCGGATCAGCAACGGATCGCGC

>P16_U1346_wzi455

ATGATAAAAATTGCGCGCATTGCCGTGACGCTGGGCTTGCTTTCCACACTGGGAGCTCAG

GCTTACGCGGCCGGGTTAGTAGTAAATGATAACGATCTGCGTAACGACCTTGCCTGGCTT

TCCGATCGCGGGGTCATCCATCTGAGCCTGTCGACCTGGCCGCTGAGCCAGGAAGAGATT

ACCCGGGCGCTAAAAAAGGCCAAACCTTCTTATTCTTCTGAGCAGGTGGTTCTGGCACGT

ATCAACCAGAGACTGTCTGCTTTAAAAGCCGATTTCCGTGTTACCGGCTATACCTCGACC

GACCAACCGGGCACTCCGCAGGGGTTTGGTCAGACGCAGCCAGCGGATAACTCGTTAGGG

CTGGCGTTCAACAATAGCGGCGAGTGGTGGGATATCCACCTTCAGGGCAACGTCGAAGGG

GGAGAGCGGATCAGCAACGGATCGCGC

>P16_S1356_wzi455

ATGATAAAAATTGCGCGCATTGCCGTGACGCTGGGCTTGCTTTCCACACTGGGAGCTCAG

GCTTACGCGGCCGGGTTAGTAGTAAATGATAACGATCTGCGTAACGACCTTGCCTGGCTT

TCCGATCGCGGGGTCATCCATCTGAGCCTGTCGACCTGGCCGCTGAGCCAGGAAGAGATT

ACCCGGGCGCTAAAAAAGGCCAAACCTTCTTATTCTTCTGAGCAGGTGGTTCTGGCACGT

ATCAACCAGAGACTGTCTGCTTTAAAAGCCGATTTCCGTGTTACCGGCTATACCTCGACC

GACCAACCGGGCACTCCGCAGGGGTTTGGTCAGACGCAGCCAGCGGATAACTCGTTAGGG

CTGGCGTTCAACAATAGCGGCGAGTGGTGGGATATCCACCTTCAGGGCAACGTCGAAGGG

GGAGAGCGGATCAGCAACGGATCGCGC

>P16_S1357_wzi455

ATGATAAAAATTGCGCGCATTGCCGTGACGCTGGGCTTGCTTTCCACACTGGGAGCTCAG

GCTTACGCGGCCGGGTTAGTAGTAAATGATAACGATCTGCGTAACGACCTTGCCTGGCTT

TCCGATCGCGGGGTCATCCATCTGAGCCTGTCGACCTGGCCGCTGAGCCAGGAAGAGATT

ACCCGGGCGCTAAAAAAGGCCAAACCTTCTTATTCTTCTGAGCAGGTGGTTCTGGCACGT

ATCAACCAGAGACTGTCTGCTTTAAAAGCCGATTTCCGTGTTACCGGCTATACCTCGACC

GACCAACCGGGCACTCCGCAGGGGTTTGGTCAGACGCAGCCAGCGGATAACTCGTTAGGG

CTGGCGTTCAACAATAGCGGCGAGTGGTGGGATATCCACCTTCAGGGCAACGTCGAAGGG

GGAGAGCGGATCAGCAACGGATCGCGC

>P16_S1358_wzi455

ATGATAAAAATTGCGCGCATTGCCGTGACGCTGGGCTTGCTTTCCACACTGGGAGCTCAG

GCTTACGCGGCCGGGTTAGTAGTAAATGATAACGATCTGCGTAACGACCTTGCCTGGCTT

TCCGATCGCGGGGTCATCCATCTGAGCCTGTCGACCTGGCCGCTGAGCCAGGAAGAGATT

ACCCGGGCGCTAAAAAAGGCCAAACCTTCTTATTCTTCTGAGCAGGTGGTTCTGGCACGT

ATCAACCAGAGACTGTCTGCTTTAAAAGCCGATTTCCGTGTTACCGGCTATACCTCGACC

GACCAACCGGGCACTCCGCAGGGGTTTGGTCAGACGCAGCCAGCGGATAACTCGTTAGGG

CTGGCGTTCAACAATAGCGGCGAGTGGTGGGATATCCACCTTCAGGGCAACGTCGAAGGG

GGAGAGCGGATCAGCAACGGATCGCGC

>P17_S394_UK2

ATGATAAAAATTGCGCGCATTGCCGTGACATTGGGCTTGCTTTCCTCACTGGGAGCCCAG

GCTTACGCGGCCGGGTTAGTAGTAAACGATAACGATCTGCGAAACGACCTTGCCTGGCTT

TCCGATCGCGGGGTCATCCATCTGAGCCTGTCGACCTGGCCGCTGAGCCAGGAAGAGATC

GCCCGGGCGCTAAAAAAAGCTAAACCGTCCTATTCTTCTGAGCAAGTGGTGCTGGCCCGT

ATCAATCAGCGACTATCTGCCTTAAAAGCCGATTTCAGGGTCACCGGCTACACCTCAACC

GACCAGCCGGGTACTCCGCAGGGTTTTGGTCAGACGCAGCCGGCAGATAACTCGTTAGGC

CTGGCGTTTAACAACAGCGGCGAGTGGTGGGATGTCCACCTTCAGGGCAACGTCGAAGGG

GGGGAGCGGATCAGCAACGGATCGCGC

>P17_S395_UK2

ATGATAAAAATTGCGCGCATTGCCGTGACATTGGGCTTGCTTTCCTCACTGGGAGCCCAG

GCTTACGCGGCCGGGTTAGTAGTAAACGATAACGATCTGCGAAACGACCTTGCCTGGCTT

TCCGATCGCGGGGTCATCCATCTGAGCCTGTCGACCTGGCCGCTGAGCCAGGAAGAGATC

GCCCGGGCGCTAAAAAAAGCTAAACCGTCCTATTCTTCTGAGCAAGTGGTGCTGGCCCGT

ATCAATCAGCGACTATCTGCCTTAAAAGCCGATTTCAGGGTCACCGGCTACACCTCAACC

GACCAGCCGGGTACTCCGCAGGGTTTTGGTCAGACGCAGCCGGCAGATAACTCGTTAGGC

CTGGCGTTTAACAACAGCGGCGAGTGGTGGGATGTCCACCTTCAGGGCAACGTCGAAGGG

GGGGAGCGGATCAGCAACGGATCGCGC

>P17_S396_UK2

ATGATAAAAATTGCGCGCATTGCCGTGACATTGGGCTTGCTTTCCTCACTGGGAGCCCAG

GCTTACGCGGCCGGGTTAGTAGTAAACGATAACGATCTGCGAAACGACCTTGCCTGGCTT

TCCGATCGCGGGGTCATCCATCTGAGCCTGTCGACCTGGCCGCTGAGCCAGGAAGAGATC

GCCCGGGCGCTAAAAAAAGCTAAACCGTCCTATTCTTCTGAGCAAGTGGTGCTGGCCCGT

ATCAATCAGCGACTATCTGCCTTAAAAGCCGATTTCAGGGTCACCGGCTACACCTCAACC

GACCAGCCGGGTACTCCGCAGGGTTTTGGTCAGACGCAGCCGGCAGATAACTCGTTAGGC

CTGGCGTTTAACAACAGCGGCGAGTGGTGGGATGTCCACCTTCAGGGCAACGTCGAAGGG

GGGGAGCGGATCAGCAACGGATCGCGC

>P18_S399_wzi50_K15K17K50K51K52

ATGATAAAAATTGCGCGCATTGCCGTGACGTTGGGTTTGCTTTCCTCCCTGGGAGCCCAG

GCTTACGCAGCCGGGTTAGTGGTAAATGACAACGACTTGCGTAACGACCTGGCCTGGCTT

TCCGATCGCGGGGTCATCCATCTGAGCCTGTCGACCTGGCCGCTCAGCCAGGAAGAGATT

GCCCGGGCGCTGAAAAAGGCCAAACCGTCCTATTCTTCTGAGCAGGTAGTTCTGGCCCGT

ATCAACCAGAGACTGTCTGCCTTAAAAGCGGATTTCCGGGTCACCGGCTACACTTCAACA

GATCAGCCGGGCACTCCGCAGGGGTTTGGCCAGACGCAGCCGGCGGATAGCTCGTTAGGC

CTGGCGTTCAACAACAGCGGCGAGTGGTGGGACGTCCACCTTCAGGGCAATGTTGAAGGG

GGAGAGCGGATCAGCAACGGATCGCGC

>P18_S400_wzi104

ATGATAAAAATTGCGCGCATTGCCGTGACGTTGGGTTTGCTTTCCTCACTGGGAGCCCAG

GCTTACGCGGCCGGGTTAGTGGTAAATGACAACGACTTACGTAACGACCTGGCCTGGCTT

TCCGATCGCGGGGTCATCCATCTGAGCCTGTCGACCTGGCCGCTGAGCCAGGAAGAGATT

GCCCGGGCGCTGAAAAAGGCCAAACCGTCCTATTCTTCTGAACAAGTGGTGCTGGCGCGT

ATTAACCAGCGACTGTCTGCCTTAAAAGCGGATTTCCGGGTCACCGGCTACACTTCAACA

GATCAGCCGGGCACTCCGCAGGGGTTTGGCCAGACGCAGCCGGCGGATAACTCGTTAGGC

CTGGCGTTCAACAACAGCGGCGAGTGGTGGGACGTCCACCTTCAGGGCAATGTTGAAGGG

GGAGAGCGGATCAGCAACGGATCGCGC

>P18_S401_wzi104

ATGATAAAAATTGCGCGCATTGCCGTGACGTTGGGTTTGCTTTCCTCACTGGGAGCCCAG

GCTTACGCGGCCGGGTTAGTGGTAAATGACAACGACTTACGTAACGACCTGGCCTGGCTT

TCCGATCGCGGGGTCATCCATCTGAGCCTGTCGACCTGGCCGCTGAGCCAGGAAGAGATT

GCCCGGGCGCTGAAAAAGGCCAAACCGTCCTATTCTTCTGAACAAGTGGTGCTGGCGCGT

ATTAACCAGCGACTGTCTGCCTTAAAAGCGGATTTCCGGGTCACCGGCTACACTTCAACA

GATCAGCCGGGCACTCCGCAGGGGTTTGGCCAGACGCAGCCGGCGGATAACTCGTTAGGC

CTGGCGTTCAACAACAGCGGCGAGTGGTGGGACGTCCACCTTCAGGGCAATGTTGAAGGG

GGAGAGCGGATCAGCAACGGATCGCGC

>P19_S402_wzi108_K80

ATGATAAAAATTGCGCGCATTGCCGTGACGTTGGGTTTGCTTTCCTCCCTGGGAGCCCAG

GCTTACGCAGCCGGGTTAGTGGTAAATGACAACGACTTGCGTAACGACCTGGCTTGGCTT

TCCGATCGCGGGGTCATCCATCTGAGCCTGTCGACCTGGCCGCTGAGCCAGGAAGAGATT

GCCCGGGCGCTGAAAAAGGCCAAACCGTCCTATTCTTCTGAGCAGGTAGTTCTGGCCCGT

ATCAACCAGAGACTGTCTGCCTTAAAAGCGGATTTCCGGGTCACCGGCTACACTTCAACA

GATCAGCCGGGCACTCCGCAGGGGTTTGGCCAGACGCAGCCGGCGGATAACTCGTTAGGC

CTGGCGTTCAACAACAGCGGCGAGTGGTGGGACGTCCACCTTCAGGGCAATGTTGAAGGG

GGAGAGCGGATCAGCAACGGATCGCGC

>P19_S403_wzi148_K30

ATGATAAAAATTGCGCGCATTGCCGTGACGTTGGGTTTGCTTTCCTCCCTGGGAGTCCAG

GCTTACGCGGCCGGGTTAGTGGTAAATGACAACGACTTGCGTAACGATCTGGCCTGGCTT

TCCGATCGCGGGGTCATCCATCTGAGCCTGTCGACCTGGCCGCTGAGCCAGGAAGAGATC

GCCCGGGCACTGAAAAAGGCTAAACCGTCCTATTCTTCTGAACAAGTGGTGCTGGCGCGT

ATCAACCAGCGACTGTCTGCTTTGAAAGCCGATTTTCGGGTCACCGGCTACACTTCAACC

GACAAGCCGGGCACTCCGCAGGGGTTTGGCCAGACGCAGCCGGCAGATAACTCGTTAGGC

CTGGCGTTCAACAACAGCGGCGAGTGGTGGGATGTCCACCTCCAGGGCAACGTCGAAGGA

GGGGAGCGGATCAGCAACGGGTCGCGC

>P19_S404_wzi108_K80

ATGATAAAAATTGCGCGCATTGCCGTGACGTTGGGTTTGCTTTCCTCCCTGGGAGCCCAG

GCTTACGCAGCCGGGTTAGTGGTAAATGACAACGACTTGCGTAACGACCTGGCTTGGCTT

TCCGATCGCGGGGTCATCCATCTGAGCCTGTCGACCTGGCCGCTGAGCCAGGAAGAGATT

GCCCGGGCGCTGAAAAAGGCCAAACCGTCCTATTCTTCTGAGCAGGTAGTTCTGGCCCGT

ATCAACCAGAGACTGTCTGCCTTAAAAGCGGATTTCCGGGTCACCGGCTACACTTCAACA

GATCAGCCGGGCACTCCGCAGGGGTTTGGCCAGACGCAGCCGGCGGATAACTCGTTAGGC

CTGGCGTTCAACAACAGCGGCGAGTGGTGGGACGTCCACCTTCAGGGCAATGTTGAAGGG

GGAGRRCGSRKMAGCAACGGATCGCGC

>P20_S413_UK3

ATGATAAAAATTGCGCGCATTGCCGTGACGCTGGGCTTGCTTTCCTCACTGGGAGCCCAG

GCTTACGCGGCCGGGTTAGTGGTAAATGATAACGATCTACGTAACGACCTTGCCTGGCTT

TCCGATCGCGGGGTCATCCATCTGAGCCTGTCGACCTGGCCGCTGAGTCAGGAAGAGATC

GCCCGGGCGCTAAAAAAGGCCAAACCGTCCTATTCTTCTGAGCAAGTGGTTCTGGCCCGT

ATCAACCAGCGACTGTCTGCTTTAAAAGCCGATTTCCGGGTCACCGGCTATACCTCGACC

GACCAGCCGGGCACCCCGCAGGGGTTTGCCCAGACGCAGCCGGCAGATAACTCGTTAGGC

CTGGCGTTTAACAATAGCGGCGAGTGGTGGGATGTCCACCTGCAGGGTAACGTCGAAGGG

GGGGAGCGGATCAGCAACGGATCGCGC

>P20_S414_UK3

ATGATAAAAATTGCGCGCATTGCCGTGACGCTGGGCTTGCTTTCCTCACTGGGAGCCCAG

GCTTACGCGGCCGGGTTAGTGGTAAATGATAACGATCTACGTAACGACCTTGCCTGGCTT

TCCGATCGCGGGGTCATCCATCTGAGCCTGTCGACCTGGCCGCTGAGTCAGGAAGAGATC

GCCCGGGCGCTAAAAAAGGCCAAACCGTCCTATTCTTCTGAGCAAGTGGTTCTGGCCCGT

ATCAACCAGCGACTGTCTGCTTTAAAAGCCGATTTCCGGGTCACCGGCTATACCTCGACC

GACCAGCCGGGCACCCCGCAGGGGTTTGCCCAGACGCAGCCGGCAGATAACTCGTTAGGC

CTGGCGTTTAACAATAGCGGCGAGTGGTGGGATGTCCACCTGCAGGGTAACGTCGAAGGG

GGGGAGCGGATCAGCAACGGATCGCGC

>P20_S415_wzi27_K27

ATGATAAAAATTGCGCGCATTGCCGTGACGTTGGGTTTGCTTTCCTCCCTGGGAGCCCAG

GCTTACGCGGCCGGGTTAGTAGTAAATGACAACGACTTGCGTAACGACCTGGCCTGGCTT

TCCGATCGTGGGGTCATCCATCTGAGCCTGTCGACTTGGCCGCTGAGCCAGGAAGAGATC

TCCCGGGCGCTAAAAAAGGCCAAACCGTCCTATTCTTCTGAGCAAGTGGTGCTGGCTCGT

ATTAACCAGCGACTGTCTGCCTTAAAAGCGGATTTCCGGGTTACCGGCTACACTTCAACC

GATCAGCCGGGCACTCCGCAGGGGTTTGGTCAGACACAGCCGGCAGATAACTCGTTAGGC

CTGGCGTTCAACAACAGCGGCGAGTGGTGGGATATCCACCTGCAGGGTAACGTCGAAGGA

GGGGAGCGGATCAGCAACGGATCGCGC

>P21_S749_wzi5_K5

ATGATAAAAATTGCGCGCATTGCCGTGACGTTGGGTTTGCTTTCCTCCCTGGGAGCCCAG

GCCTACGCGGCCGGGTTAGTGGTAAATGATAACGATCTGCGTAACGACCTGGCCTGGCTT

TCCGATCGCGGGGTCATCCATCTGAGCCTGTCGACGTGGCCGCTGAGCCAGGAAGAGATC

GCCCGTGCGTTAAAAAAGGCCAAACCGTCCTATTCTTCTGAGCAAGTGGTGCTGGCGCGT

ATTAACCAGCGGCTGTCTGCCTTAAAAGCGGATTTCCGGGTCACCGGCTACACCTCAACC

GATCAGCCGGGCACTCCGCAGGGATTTGGCCAGACGCAGCCAGCAGATAACTCGTTAGGC

CTGGCGTTCAACAACAGCGGCGAGTGGTGGGATATCCATCTCCAGKGCAACGTTGAAGGG

GGAGAGCGGATCAGCAACGGATCGCGC

>P21_S750_UK4

ATGATAAAAATTGCGCGCATTGCCGTGACGCTGGGGGTTCTTTCCTCTCTGGGAGCCCAG

GCTTACGCGGCTGGGTTAGTGGTAAATGACAACGATTTGAGAAATGACCTTGCCTGGCTT

TCCGACCGTGGAGTTATTCATCTGAGCCTGTCGACGTGGCCGCTGAGTCAGGAAGAGATC

TCCCGGGCGCTGAAAAAGGCCAAACCTTCCTATTCTTCTGAGCAAGTGGTGCTGGCTCGA

ATTAACCAGCGACTGTCTGCCTTAAAAGCCGATTTCCGGGTCACCGGCTACACTTCAACC

GATCAGCCGGGCACTCCGCAGGGGTTTGGTCAGACACAGCCGGCAGATAACTCGTTAGGC

CTGGCGTTCAACAACAGCGGCGAGTGGTGGGATGTCCACCGGCAGGGCAACGTCGAAGGG

GGTGAGCGGATCAGCAACGGATCGCGC

>P21_S751_wzi5_K5

ATGATAAAAATTGCGCGCATTGCCGTGACGTTGGGTTTGCTTTCCTCCCTGGGAGCCCAG

GCCTACGCGGCCGGGTTAGTGGTAAATGATAACGATCTGCGTAACGACCTGGCCTGGCTT

TCCGATCGCGGGGTCATCCATCTGAGCCTGTCGACGTGGCCGCTGAGCCAGGAAGAGATC

GCCCGTGCGTTAAAAAAGGCCAAACCGTCCTATTCTTCTGAGCAAGTGGTGCTGGCGCGT

ATTAACCAGCGGCTGTCTGCCTTAAAAGCGGATTTCCGGGTCACCGGCTACACCTCAACC

GATCAGCCGGGCACTCCGCAGGGATTTGGCCAGACGCAGCCAGCAGATAACTCGTTAGGC

CTGGCGTTCAACAACAGCGGCGAGTGGTGGGATATCCATCTCCAGGGCAACGTTGAAGGG

GGAGAGCGGATCAGCAACGGATCGCGC

>P22_S935_wzi24_K24

ATGATAAAAATTGCGCGCATTGCCGTGACGTTGGGTTTGCTTTCCTCCCTGGGAGCCCAG

GCTTACGCGGCCGGGTTAGTGGTAAATGACAACGACTTGCGTAACGACCTGGCCTGGCTT

TCCGATCGCGGGGTCATCCATCTGAGCCTGTCGACCTGGCCGCTGAGCCAGGAAGAGATC

TCCCGGGCGCTAAAAAAGGCCAAACCGTCCTATTCTTCTGAACAAGTGGTGCTGGCGCGT

ATTAACCAGCGCCTGTCTGCCTTAAAAGCGGATTTCCGGGTTACCGGCTACACTTCAACC

GATCAGCCGGGCACTCCGCAGGGGTTTGGTCAGACACAGCCGGCAGATAACTCGTTAGGC

CTGGCGTTCAACAACAGCGGCGAGTGGTGGGATGTCCACCTCCAGGGCAACGTCGAAGGA

GGGGAGCGGATCAGCAACGGCTCGCGC

>P22_S936_wzi24_K24

ATGATAAAAATTGCGCGCATTGCCGTGACGTTGGGTTTGCTTTCCTCCCTGGGAGCCCAG

GCTTACGCGGCCGGGTTAGTGGTAAATGACAACGACTTGCGTAACGACCTGGCCTGGCTT

TCCGATCGCGGGGTCATCCATCTGAGCCTGTCGACCTGGCCGCTGAGCCAGGAAGAGATC

TCCCGGGCGCTAAAAAAGGCCAAACCGTCCTATTCTTCTGAACAAGTGGTGCTGGCGCGT

ATTAACCAGCGCCTGTCTGCCTTAAAAGCGGATTTCCGGGTTACCGGCTACACTTCAACC

GATCAGCCGGGCACTCCGCAGGGGTTTGGTCAGACACAGCCGGCAGATAACTCGTTAGGC

CTGGCGTTCAACAACAGCGGCGAGTGGTGGGATGTCCACCTCCAGGGCAACGTCGAAGGA

GGGGAGCGGATCAGCAACGGCTCGCGC

>P22_S937_UK5

ATGATAAAAATTGCGCGCATTGCCGTGACGTTGGGTTTGCTTTCCTCCCTGGGAGCCCAG

GCTTTCGCGGCCGGGTTAGTGGTAAATGACAACGACTTGCGTAACGACCTGGCCTGGCTT

TCCGATCGCGGGGTCATCCATCTGAGCCTGTCGACCTGGCCGCTGAGCCAGGAAGAGATC

TCCCGGGCGCTAAAAAAAGCCAAACCGTCCTATTCTTCTGAACAAGTGGTGCTGGCGCGT

ATTAACCAGCGACTGTCTGCCTTAAAAGCCGATTTCCGGGTCACCGGCTACACTTCAACC

GATCAGCCGGGCACTCCACAGGGGTTTGGTCAGACACAGCCGGCGGATAACTCGTTAGGC

CTGGCGTTCAACAACAGCGGCGAGTGGTGGGATGTTCACCTGCAGGGCAACGTCGAAGGG

GGGGAGCGGATCAGCAACGGATCGCGC

>P23_S1449_wzi100_K10

ATGATAAAAATTGCGCGCATTGCCGTGACGTTGGGTTTGCTTTCCTCCCTGGGAGCCCAG

GCTTACGCGGCCGGGTTAGTAGTAAATGACAACGACTTGCGTAACGACCTGGCCTGGCTT

TCCGATCGTGGGGTCATTCATCTGAGCCTGTCGACGTGGCCGCTAAGCCAGGAAGAGATC

GCCCGGGCGCTGAAAAAGGCCAAGCCTTCCTATTCTTCTGAGCAAGTGGTCCTTGCTCGT

ATTAACCAGCGACTGTCTGCCTTAAAAGCGGATTTCCGGGTTACCGGCTACACTTCAACC

GATCAGCCGGGCACTCCGCAGGGGTTTGGTCAGACACAGCCGGCAGATAACTCGTTAGGC

CTGGCGTTCAACAACAGCGGCGAGTGGTGGGATATCCACCTCCAGGGCAACGTCGAAGGA

GGGGAGCGGATCAGCAACGGATCGCGC

>P23_S1450_wzi100_K10

ATGATAAAAATTGCGCGCATTGCCGTGACGTTGGGTTTGCTTTCCTCCCTGGGAGCCCAG

GCTTACGCGGCCGGGTTAGTAGTAAATGACAACGACTTGCGTAACGACCTGGCCTGGCTT

TCCGATCGTGGGGTCATTCATCTGAGCCTGTCGACGTGGCCGCTAAGCCAGGAAGAGATC

GCCCGGGCGCTGAAAAAGGCCAAGCCTTCCTATTCTTCTGAGCAAGTGGTCCTTGCTCGT

ATTAACCAGCGACTGTCTGCCTTAAAAGCGGATTTCCGGGTTACCGGCTACACTTCAACC

GATCAGCCGGGCACTCCGCAGGGGTTTGGTCAGACACAGCCGGCAGATAACTCGTTAGGC

CTGGCGTTCAACAACAGCGGCGAGTGGTGGGATATCCACCTCCAGGGCAACGTCGAAGGA

GGGGAGCGGATCAGCAACGGATCGCGC

>P23_S1451_wzi100_K10

ATGATAAAAATTGCGCGCATTGCCGTGACGTTGGGTTTGCTTTCCTCCCTGGGAGCCCAG

GCTTACGCGGCCGGGTTAGTAGTAAATGACAACGACTTGCGTAACGACCTGGCCTGGCTT

TCCGATCGTGGGGTCATTCATCTGAGCCTGTCGACGTGGCCGCTAAGCCAGGAAGAGATC

GCCCGGGCGCTGAAAAAGGCCAAGCCTTCCTATTCTTCTGAGCAAGTGGTCCTTGCTCGT

ATTAACCAGCGACTGTCTGCCTTAAAAGCGGATTTCCGGGTTACCGGCTACACTTCAACC

GATCAGCCGGGCACTCCGCAGGGGTTTGGTCAGACACAGCCGGCAGATAACTCGTTAGGC

CTGGCGTTCAACAACAGCGGCGAGTGGTGGGATATCCACCTCCAGGGCAACGTCGAAGGA

GGGGAGCGGATCAGCAACGGATCGCGC

>P24_S1574_wzi31_K31

ATGATAAAAATTGCGCGCATTGCCGTGACGTTGGGTTTGCTTTCCTCCCTGGGAGCCCAG

GCTTACGCGGCCGGGTTAGTGGTAAATGACAACGACTTGCGTAACGACCTGGCCTGGCTT

TCCGATCGCGGGGTCATCCATCTGAGCCTGTCGACCTGGCCGCTCAGCCAGGAAGAGATC

GCCCGGGCACTGAAAAAGGCTAAACCGTCCTATTCTTCTGAACAAGTGGTGCTGGCGCGT

ATTAACCAGCGACTGTCTGCCTTAAAAGCCGATTTCCGGGTCACCGGCTACACTTCAACC

GATCAGCCGGGCACTCCACAGGGGTTTGGCCAGACGCAACCGGCGGATAATTCGTTAGGG

CTGGCGTTCAATAACAGCGGTGAGTGGTGGGATGTCCACCTGCAGGGCAACGTCGAAGGA

GGGGAGCGGATCAGCAACGGCTCGCGC

>P24_S1575_wzi31_K31

ATGATAAAAATTGCGCGCATTGCCGTGACGTTGGGTTTGCTTTCCTCCCTGGGAGCCCAG

GCTTACGCGGCCGGGTTAGTGGTAAATGACAACGACTTGCGTAACGACCTGGCCTGGCTT

TCCGATCGCGGGGTCATCCATCTGAGCCTGTCGACCTGGCCGCTCAGCCAGGAAGAGATC

GCCCGGGCACTGAAAAAGGCTAAACCGTCCTATTCTTCTGAACAAGTGGTGCTGGCGCGT

ATTAACCAGCGACTGTCTGCCTTAAAAGCCGATTTCCGGGTCACCGGCTACACTTCAACC

GATCAGCCGGGCACTCCACAGGGGTTTGGCCAGACGCAACCGGCGGATAATTCGTTAGGG

CTGGCGTTCAATAACAGCGGTGAGTGGTGGGATGTCCACCTGCAGGGCAACGTCGAAGGA

GGGGAGCGGATCAGCAACGGCTCGCGC

>P25_S1540_UK6

ATGATAAAAATTGCGCGCATTGCCGTGACGTTGGGTTTGCTTTCCTCACTGGGAGCCCAG

GCTTACGCGGCCGGGTTAGTGGTAAATGACAACGATTTGCGTAACGACCTGGCCTGGCTT

TCCGATCGCGGAGTCATCCATCTGAGCCTGTCGACGTGGCCGCTGAGCCAGGAAGAGATC

GCCCGGACGCTGAAAAAGGCCAAACCGTCCTATTCTTCTGAGCAAGTGGTGCTGGCTCGT

ATCAACCAGCGACTGTCTGCCTTAAAAGCCGATTTCCGGGTCACCGGCTACACTTCAACC

GACCAGCCGGGCACTCCGCAGGGGTTTGGCCAGACGCAGCCGGCGGATAACTCGTTAGGC

CTGGCGTTCAACAACAGCGGTGAGTGGTGGGATGTCCACCTGCAGGGCAACGTCGAAGGA

GGGGAGCGGATCAGCAACGGCTCGCGC

>P25_S1541_UK6

ATGATAAAAATTGCGCGCATTGCCGTGACGTTGGGTTTGCTTTCCTCACTGGGAGCCCAG

GCTTACGCGGCCGGGTTAGTGGTAAATGACAACGATTTGCGTAACGACCTGGCCTGGCTT

TCCGATCGCGGAGTCATCCATCTGAGCCTGTCGACGTGGCCGCTGAGCCAGGAAGAGATC

GCCCGGACGCTGAAAAAGGCCAAACCGTCCTATTCTTCTGAGCAAGTGGTGCTGGCTCGT

ATCAACCAGCGACTGTCTGCCTTAAAAGCCGATTTCCGGGTCACCGGCTACACTTCAACC

GACCAGCCGGGCACTCCGCAGGGGTTTGGCCAGACGCAGCCGGCGGATAACTCGTTAGGC

CTGGCGTTCAACAACAGCGGTGAGTGGTGGGATGTCCACCTGCAGGGCAACGTCGAAGGA

GGGGAGCGGATCAGCAACGGCTCGCGC

>P25_S1542_UK6

ATGATAAAAATTGCGCGCATTGCCGTGACGTTGGGTTTGCTTTCCTCACTGGGAGCCCAG

GCTTACGCGGCCGGGTTAGTGGTAAATGACAACGATTTGCGTAACGACCTGGCCTGGCTT

TCCGATCGCGGAGTCATCCATCTGAGCCTGTCGACGTGGCCGCTGAGCCAGGAAGAGATC

GCCCGGACGCTGAAAAAGGCCAAACCGTCCTATTCTTCTGAGCAAGTGGTGCTGGCTCGT

ATCAACCAGCGACTGTCTGCCTTAAAAGCCGATTTCCGGGTCACCGGCTACACTTCAACC

GACCAGCCGGGCACTCCGCAGGGGTTTGGCCAGACGCAGCCGGCGGATAACTCGTTAGGC

CTGGCGTTCAACAACAGCGGTGAGTGGTGGGATGTCCACCTGCAGGGCAACGTCGAAGGA

GGGGAGCGGATCAGCAACGGCTCGCGC

>P26_S1650_wzi24_K24

ATGATAAAAATTGCGCGCATTGCCGTGACGTTGGGTTTGCTTTCCTCCCTGGGAGCCCAG

GCTTACGCGGCCGGGTTAGTGGTAAATGACAACGACTTGCGTAACGACCTGGCCTGGCTT

TCCGATCGCGGGGTCATCCATCTGAGCCTGTCGACCTGGCCGCTGAGCCAGGAAGAGATC

TCCCGGGCGCTAAAAAAGGCCAAACCGTCCTATTCTTCTGAACAAGTGGTGCTGGCGCGT

ATTAACCAGCGCCTGTCTGCCTTAAAAGCGGATTTCCGGGTTACCGGCTACACTTCAACC

GATCAGCCGGGCACTCCGCAGGGGTTTGGTCAGACACAGCCGGCAGATAACTCGTTAGGC

CTGGCGTTCAACAACAGCGGCGAGTGGTGGGATGTCCACCTCCAGGGCAACGTCGAAGGA

GGGGAGCGGATCAGCAACGGCTCGCGC

>P26_S1651_wzi24_K24

ATGATAAAAATTGCGCGCATTGCCGTGACGTTGGGTTTGCTTTCCTCCCTGGGAGCCCAG

GCTTACGCGGCCGGGTTAGTGGTAAATGACAACGACTTGCGTAACGACCTGGCCTGGCTT

TCCGATCGCGGGGTCATCCATCTGAGCCTGTCGACCTGGCCGCTGAGCCAGGAAGAGATC

TCCCGGGCGCTAAAAAAGGCCAAACCGTCCTATTCTTCTGAACAAGTGGTGCTGGCGCGT

ATTAACCAGCGCCTGTCTGCCTTAAAAGCGGATTTCCGGGTTACCGGCTACACTTCAACC

GATCAGCCGGGCACTCCGCAGGGGTTTGGTCAGACACAGCCGGCAGATAACTCGTTAGGC

CTGGCGTTCAACAACAGCGGCGAGTGGTGGGATGTCCACCTCCAGGGCAACGTCGAAGGA

GGGGAGCGGATCAGCAACGGCTCGCGC

>P26_S1652_wzi24_K24

ATGATAAAAATTGCGCGCATTGCCGTGACGTTGGGTTTGCTTTCCTCCCTGGGAGCCCAG

GCTTACGCGGCCGGGTTAGTGGTAAATGACAACGACTTGCGTAACGACCTGGCCTGGCTT

TCCGATCGCGGGGTCATCCATCTGAGCCTGTCGACCTGGCCGCTGAGCCAGGAAGAGATC

TCCCGGGCGCTAAAAAAGGCCAAACCGTCCTATTCTTCTGAACAAGTGGTGCTGGCGCGT

ATTAACCAGCGCCTGTCTGCCTTAAAAGCGGATTTCCGGGTTACCGGCTACACTTCAACC

GATCAGCCGGGCACTCCGCAGGGGTTTGGTCAGACACAGCCGGCAGATAACTCGTTAGGC

CTGGCGTTCAACAACAGCGGCGAGTGGTGGGATGTCCACCTCCAGGGCAACGTCGAAGGA

GGGGAGCGGATCAGCAACGGCTCGCGC

>P27_S1173_UK7

ATGATAAAAATTGCGCGCATTGCCGTGACGCTGGGCTTGCTTTCCTCACTGGGAGCCCAG

GCTTACGCGGCCGGGTTAGTAGTAAATGATAACGATCTGCGAAACGACCTTGCCTGGCTT

TCCGATCGCGGGGTCATCCATCTGAGCCTGTCGACCTGGCCGCTTAGCCAGGAAGAGATC

GCCCGGGCGCTAAAAAAAGCTAAACCGTCCTATTCTTCTGAGCAAGTAGTGCTGGCCCGT

ATCAACCAGCGACTGTCTGCTTTAAAAGCCGATTTCCGGGTCACCGGCTACACCTCAACC

GACCAGCCTGGCACCCCGCAGGGGTTTGGCCAGACGCAGCCGGCAGATAACTCGTTAGGC

CTGGCGTTTAACAACAGCGGCGAGTGGTGGGACGTTCACCTCCAGGGCAACGTTGAAGGG

GGAGAGCGAATCAGTAACGGATCGCGC

>P27_S1174_UK7

ATGATAAAAATTGCGCGCATTGCCGTGACGCTGGGCTTGCTTTCCTCACTGGGAGCCCAG

GCTTACGCGGCCGGGTTAGTAGTAAATGATAACGATCTGCGAAACGACCTTGCCTGGCTT

TCCGATCGCGGGGTCATCCATCTGAGCCTGTCGACCTGGCCGCTTAGCCAGGAAGAGATC

GCCCGGGCGCTAAAAAAAGCTAAACCGTCCTATTCTTCTGAGCAAGTAGTGCTGGCCCGT

ATCAACCAGCGACTGTCTGCTTTAAAAGCCGATTTCCGGGTCACCGGCTACACCTCAACC

GACCAGCCTGGCACCCCGCAGGGGTTTGGCCAGACGCAGCCGGCAGATAACTCGTTAGGC

CTGGCGTTTAACAACAGCGGCGAGTGGTGGGACGTTCACCTCCAGGGCAACGTTGAAGGG

GGAGAGCGAATCAGTAACGGATCGCGC

>P27_S1175_UK7

ATGATAAAAATTGCGCGCATTGCCGTGACGCTGGGCTTGCTTTCCTCACTGGGAGCCCAG

GCTTACGCGGCCGGGTTAGTAGTAAATGATAACGATCTGCGAAACGACCTTGCCTGGCTT

TCCGATCGCGGGGTCATCCATCTGAGCCTGTCGACCTGGCCGCTTAGCCAGGAAGAGATC

GCCCGGGCGCTAAAAAAAGCTAAACCGTCCTATTCTTCTGAGCAAGTAGTGCTGGCCCGT

ATCAACCAGCGACTGTCTGCTTTAAAAGCCGATTTCCGGGTCACCGGCTACACCTCAACC

GACCAGCCTGGCACCCCGCAGGGGTTTGGCCAGACGCAGCCGGCAGATAACTCGTTAGGC

CTGGCGTTTAACAACAGCGGCGAGTGGTGGGACGTTCACCTCCAGGGCAACGTTGAAGGG

GGAGAGCGAATCAGTAACGGATCGCGC

>P28_S889_UK8

ATGATAAAAATTGCGCGCATTGCCGTGACGTTGGGTTTGCTTTCCTCACTGGGAGCCCAG

GCTTACGCGGCTGGGTTAGTGGTAAATGACAACGACTTACGTAACGACCTAGCCTGGCTT

TCCGATCGCGGGGTCATCCATCTGAGCCTGTCGACCTGGCCGCTGAGCCAGGAAGAGATC

GCCCGGGCGCTAAAGAAGGCCAAGCCGTCTTATTCTTCTGAGCAAGTAGTTCTGGCCCGT

ATCAACCAGCGACTGTCTGCCTTAAAAGCGGATTTCCGGGTCACCGGCTACACTTCAACC

GATCAGCCGGGCACTCCGCAGGGGTTTGGCCAGACGCAGCCGGCGGATAATTCGTTAGGC

CTGGCGTTCAATAACAGCGGTGAGTGGTGGGATGTCCACCTGCAGGGCAACGTCGAAGGA

GGGGAGCGGATCAGCAACGGCTCGCGC

>P29_S503_UK9

ATGATAAAAATTGCGCGCATTGCCGTGACGTTAGGTTTGCTTTCCTCACTGGGAGCCCAG

GCTTACGCGGCCGGGTTAGTAGTAAATGATAATGATCTGCGTAACGACCTGGCCTGGCTT

TCCGATCGCGGGGTCATCCATCTGAGCCTGTCGACGTGGCCGCTGAGCCAGGAAGAGATC

GCCCGGGCGCTGAAAAAGGCCAAACCTTCCTACTCTTCTGAGCAAGTGGTGCTGGCGCGT

ATCAACCAGCGACTGTCTGCCTTAAAAGCGGATTTCCGGGTCACCGGCTACACTTCAACC

GATCAGCCGGGCACTCCGCAGGGGTTTGGTCAGACACAGCCGGCGGATAACTCGTTAGGC

CTGGCATTCAACAACAGCGGCGAGTGGTGGGATATCCATCTCCAGGGCAACGTTGAAGGG

GGAGAGCGGATCAGCAACGGCTCGCGT

>P29_S504_UK9

ATGATAAAAATTGCGCGCATTGCCGTGACGTTAGGTTTGCTTTCCTCACTGGGAGCCCAG

GCTTACGCGGCCGGGTTAGTAGTAAATGATAATGATCTGCGTAACGACCTGGCCTGGCTT

TCCGATCGCGGGGTCATCCATCTGAGCCTGTCGACGTGGCCGCTGAGCCAGGAAGAGATC

GCCCGGGCGCTGAAAAAGGCCAAACCTTCCTACTCTTCTGAGCAAGTGGTGCTGGCGCGT

ATCAACCAGCGACTGTCTGCCTTAAAAGCGGATTTCCGGGTCACCGGCTACACTTCAACC

GATCAGCCGGGCACTCCGCAGGGGTTTGGTCAGACACAGCCGGCGGATAACTCGTTAGGC

CTGGCATTCAACAACAGCGGCGAGTGGTGGGATATCCATCTCCAGGGCAACGTTGAAGGG

GGAGAGCGGATCAGCAACGGCTCGCGT

>P29_S505_UK9

ATGATAAAAATTGCGCGCATTGCCGTGACGTTAGGTTTGCTTTCCTCACTGGGAGCCCAG

GCTTACGCGGCCGGGTTAGTAGTAAATGATAATGATCTGCGTAACGACCTGGCCTGGCTT

TCCGATCGCGGGGTCATCCATCTGAGCCTGTCGACGTGGCCGCTGAGCCAGGAAGAGATC

GCCCGGGCGCTGAAAAAGGCCAAACCTTCCTACTCTTCTGAGCAAGTGGTGCTGGCGCGT

ATCAACCAGCGACTGTCTGCCTTAAAAGCGGATTTCCGGGTCACCGGCTACACTTCAACC

GATCAGCCGGGCACTCCGCAGGGGTTTGGTCAGACACAGCCGGCGGATAACTCGTTAGGC

CTGGCATTCAACAACAGCGGCGAGTGGTGGGATATCCATCTCCAGGGCAACGTTGAAGGG

GGAGAGCGGATCAGCAACGGCTCGCGT

>P30_S424_wzi93_K60

ATGATAAAAATTGCGCGCATTGCCGTGACGTTGGGTTTGCTTTCCTCCCTGGGAGCCCAG

GCTTTCGCGGCCGGGTTAGTGGTAAATGACAACGACTTGCGTAACGACCTGGCCTGGCTT

TCCGATCGCGGGGTCATCCATCTGAGCCTGTCGACTTGGCCGCTGAGCCAGGAAGAAATC

TCCCGGGCGCTAAAAAAGGCCAAACCTTCCTACTCTTCTGAGCAAGTGGTGCTGGCGCGT

ATCAACCAGCGACTGTCTGCCTTAAAAGCGGATTTCCGGGTCACCGGCTACACTTCAACC

GATCAGCCGGGCACTCCGCAGGGGTTTGGTCAGACACAGCCGGCGGATAACTCGTTAGGC

CTGGCATTCAACAACAGCGGCGAGTGGTGGGATATCCATTTCCAGGGCAACGTTGAAGGG

GGAGAGCGGATCAGCAACGGATCGCGC

>P30_S425_wzi93_K60

ATGATAAAAATTGCGCGCATTGCCGTGACGTTGGGTTTGCTTTCCTCCCTGGGAGCCCAG

GCTTTCGCGGCCGGGTTAGTGGTAAATGACAACGACTTGCGTAACGACCTGGCCTGGCTT

TCCGATCGCGGGGTCATCCATCTGAGCCTGTCGACTTGGCCGCTGAGCCAGGAAGAAATC

TCCCGGGCGCTAAAAAAGGCCAAACCTTCCTACTCTTCTGAGCAAGTGGTGCTGGCGCGT

ATCAACCAGCGACTGTCTGCCTTAAAAGCGGATTTCCGGGTCACCGGCTACACTTCAACC

GATCAGCCGGGCACTCCGCAGGGGTTTGGTCAGACACAGCCGGCGGATAACTCGTTAGGC

CTGGCATTCAACAACAGCGGCGAGTGGTGGGATATCCATTTCCAGGGCAACGTTGAAGGG

GGAGAGCGGATCAGCAWCGGATCGCGC

>P30_S426_wzi93_K60

ATGATAAAAATTGCGCGCATTGCCGTGACGTTGGGTTTGCTTTCCTCCCTGGGAGCCCAG

GCTTTCGCGGCCGGGTTAGTGGTAAATGACAACGACTTGCGTAACGACCTGGCCTGGCTT

TCCGATCGCGGGGTCATCCATCTGAGCCTGTCGACTTGGCCGCTGAGCCAGGAAGAAATC

TCCCGGGCGCTAAAAAAGGCCAAACCTTCCTACTCTTCTGAGCAAGTGGTGCTGGCGCGT

ATCAACCAGCGACTGTCTGCCTTAAAAGCGGATTTCCGGGTCACCGGCTACACTTCAACC

GATCAGCCGGGCACTCCGCAGGGGTTTGGTCAGACACAGCCGGCGGATAACTCGTTAGGC

CTGGCATTCAACAACAGCGGCGAGTGGTGGGATATCCATTTCCAGGGCAACGTTGAAGGG

GGAGAGCGGATCAGCAACGGATCGCGC

>P31_S886_wzi91_K14

ATGATAAAAATTGCGCACATTGCCGTGACGTTGGGTTTGCTTTCCTCACTGGGAGCCCAG

GCTTACGCGGCCGGGTTAGTGGTAAATGACAACGACTTGCGTAACGACCTGGCCTGGCTT

TCCGATCGCGGGGTCATCCATCTGAGCCTGTCGACCTGGCCGCTGAGCCAGGAAGAGATC

TCCCGGGCGCTAAAAAAGGCCAAACCGTCCTATTCTTCTGAGCAAGTGGTGCTGGCGCGT

ATTAACCAGCGACTGTCTGCCTTAAAAGCGGATTTCCGGGTCACCGGCTACACTTCAACC

GATCAGCCGGGCACTCCGCAGGGGTTTGGTCAGACACAGCCGGCGGATAACTCGTTAGGC

CTGGCATTCAACAACAGCGGCGAGTGGTGGGATATCCATCTCCAGGGCAACGTTGAAGGG

GGAGAGCGGATCAGCAACGGATCGCGC

>P31_S887_wzi91_K14

ATGATAAAAATTGCGCACATTGCCGTGACGTTGGGTTTGCTTTCCTCACTGGGAGCCCAG

GCTTACGCGGCCGGGTTAGTGGTAAATGACAACGACTTGCGTAACGACCTGGCCTGGCTT

TCCGATCGCGGGGTCATCCATCTGAGCCTGTCGACCTGGCCGCTGAGCCAGGAAGAGATC

TCCCGGGCGCTAAAAAAGGCCAAACCGTCCTATTCTTCTGAGCAAGTGGTGCTGGCGCGT

ATTAACCAGCGACTGTCTGCCTTAAAAGCGGATTTCCGGGTCACCGGCTACACTTCAACC

GATCAGCCGGGCACTCCGCAGGGGTTTGGTCAGACACAGCCGGCGGATAACTCGTTAGGC

CTGGCATTCAACAACAGCGGCGAGTGGTGGGATATCCATCTCCAGGGCAACGTTGAAGGG

GGAGAGCGGATCAGCAACGGATCGCGC

>P31_S888_wzi91_K14

ATGATAAAAATTGCGCACATTGCCGTGACGTTGGGTTTGCTTTCCTCACTGGGAGCCCAG

GCTTACGCGGCCGGGTTAGTGGTAAATGACAACGACTTGCGTAACGACCTGGCCTGGCTT

TCCGATCGCGGGGTCATCCATCTGAGCCTGTCGACCTGGCCGCTGAGCCAGGAAGAGATC

TCCCGGGCGCTAAAAAAGGCCAAACCGTCCTATTCTTCTGAGCAAGTGGTGCTGGCGCGT

ATTAACCAGCGACTGTCTGCCTTAAAAGCGGATTTCCGGGTCACCGGCTACACTTCAACC

GATCAGCCGGGCACTCCGCAGGGGTTTGGTCAGACACAGCCGGCGGATAACTCGTTAGGC

CTGGCATTCAACAACAGCGGCGAGTGGTGGGATATCCATCTCCAGGGCAACGTTGAAGGG

GGAGAGCGGATCAGCAACGGATCGCGC

>P32_S968_wzi96_K38

ATGATAAAAATTGCGCGCATTGCCGTGACGTTGGGTTTGCTTTCCTCACTGGGAGCCCAG

GCTTACGCGGCCGGGTTAGTGGTAAATGACAACGACTTGCGTAACGACCTGGCCTGGCTT

TCCGATCGCGGGGTCATCCATCTGAGCCTGTCGACGTGGCCGCTGAGCCAGGAAGAGATC

GCCCGGGCGCTGAAAAAGGCCAAACCTTCCTATTCTTCTGAGCAAGTGGTGCTGGCGCGT

ATTAACCAGCGACTGTCTGCCTTAAAAGCGGATTTCCGGGTCACCGGCTACACTTCAACC

GATCAGCCAGGCACTCCGCAGGGGTTTGGTCAGACGCAGTCGGCGGATAATTCGTTAGGC

CTGGCGTTCAACAACAGCGGCGAGTGGTGGGACGTCCACCTTCAGGGCAATGTTGAAGGG

GGAGAGCGGATCAGCAACGGATCGCGC

>P32_S969_wzi96_K38

ATGATAAAAATTGCGCGCATTGCCGTGACGTTGGGTTTGCTTTCCTCACTGGGAGCCCAG

GCTTACGCGGCCGGGTTAGTGGTAAATGACAACGACTTGCGTAACGACCTGGCCTGGCTT

TCCGATCGCGGGGTCATCCATCTGAGCCTGTCGACGTGGCCGCTGAGCCAGGAAGAGATC

GCCCGGGCGCTGAAAAAGGCCAAACCTTCCTATTCTTCTGAGCAAGTGGTGCTGGCGCGT

ATTAACCAGCGACTGTCTGCCTTAAAAGCGGATTTCCGGGTCACCGGCTACACTTCAACC

GATCAGCCAGGCACTCCGCAGGGGTTTGGTCAGACGCAGTCGGCGGATAATTCGTTAGGC

CTGGCGTTCAACAACAGCGGCGAGTGGTGGGACGTCCACCTTCAGGGCAATGTTGAAGGG

GGAGAGCGGATCAGCAACGGATCGCGC

>P32_S970_wzi96_K38

ATGATAAAAATTGCGCGCATTGCCGTGACGTTGGGTTTGCTTTCCTCACTGGGAGCCCAG

GCTTACGCGGCCGGGTTAGTGGTAAATGACAACGACTTGCGTAACGACCTGGCCTGGCTT

TCCGATCGCGGGGTCATCCATCTGAGCCTGTCGACGTGGCCGCTGAGCCAGGAAGAGATC

GCCCGGGCGCTGAAAAAGGCCAAACCTTCCTATTCTTCTGAGCAAGTGGTGCTGGCGCGT

ATTAACCAGCGACTGTCTGCCTTAAAAGCGGATTTCCGGGTCACCGGCTACACTTCAACC

GATCAGCCAGGCACTCCGCAGGGGTTTGGTCAGACGCAGTCGGCGGATAATTCGTTAGGC

CTGGCGTTCAACAACAGCGGCGAGTGGTGGGACGTCCACCTTCAGGGCAATGTTGAAGGG

GGAGAGCGGATCAGCAACGGATCGCGC

>P33_S687_UK10

ATGATAAAAATTGCGCGCATTGCCGTGACATTGGGCTTGCTTTCCTCACTGGGAGCCCAG

GCTTACGCGGCCGGGTTAGTAGTAAATGATAACGATCTGCGAAACGACCTTGCCTGGCTT

TCCGATCGCGGGGTTATCCGTCTGAGCCTGTCGACCTGGCCGCTGAGCCAGGAAGAGATC

ACCCGGGCGTTAAAAAAAGCTAAACCGTCCTATTCTTCTGAGCAAGTGGTGCTGGCCCGT

ATCAATCAGCGACTGTCAGCGTTAAAAGCCGATTTTCGGGTCACCGGCTACGCCTCAACC

GACCAGCCGGGCACTCCGCAGGGGTTTGGTCAGACGCAGCCGGCAGATAACTCCTTAGGC

CTGGCGTTCAACAACAGCGGCGAGTGGTGGGATGTCCACCTCCAGGGCAACGTTGAGGGG

GGAGAGCGGATCAGCAACGGTTCGCGC

>P34_S1839_UK11

ATGATAAAAATTGCGCGCATTGCCGTGACGCTGGGGTTGCTTTCCTCTCTGGGAGCCCAG

GTTTACGCGGCTGGGTTAGTGGTAAATGACAACGATTTGCGAAATGACCTTGCCTGGCTT

TCCGACCGTGGGGTCATTCATCTGAGCCTGTCGACGTGGCCGCTGAGCCAGGAAGAGATC

GCCCGGGCGCTGAAAAAGGCCAAGCCTTCCTATTCTTCTGAGCAAGTGGTCCTGGCTCGT

ATTAACCAGCGACTGTCTGGCTTAAAAGCCGATTTCCGGGTCACCGGTTACACTTCAACC

GACCAGCCGGGCACTCCGCAGGGGTTTGGCCAGACGCAGCCGGCGGATAACTCGTTAGGC

CTGGCATTCAACAACAGTGGCGAGTGGTGGGACGTACACCTTCAGGGCAACGTTGAAGGG

GGAGAACAGATCAGCAACGGATCGCGC

>P34_S1840_UK11

ATGATAAAAATTGCGCGCATTGCCGTGACGCTGGGGTTGCTTTCCTCTCTGGGAGCCCAG

GTTTACGCGGCTGGGTTAGTGGTAAATGACAACGATTTGCGAAATGACCTTGCCTGGCTT

TCCGACCGTGGGGTCATTCATCTGAGCCTGTCGACGTGGCCGCTGAGCCAGGAAGAGATC

GCCCGGGCGCTGAAAAAGGCCAAGCCTTCCTATTCTTCTGAGCAAGTGGTCCTGGCTCGT

ATTAACCAGCGACTGTCTGGCTTAAAAGCCGATTTCCGGGTCACCGGTTACACTTCAACC

GACCAGCCGGGCACTCCGCAGGGGTTTGGCCAGACGCAGCCGGCGGATAACTCGTTAGGC

CTGGCATTCAACAACAGTGGCGAGTGGTGGGACGTACACCTTCAGGGCAACGTTGAAGGG

GGAGAACAGATCAGCAACGGATCGCGC

>P34_S1841_UK11

ATGATAAAAATTGCGCGCATTGCCGTGACGCTGGGGTTGCTTTCCTCTCTGGGAGCCCAG

GTTTACGCGGCTGGGTTAGTGGTAAATGACAACGATTTGCGAAATGACCTTGCCTGGCTT

TCCGACCGTGGGGTCATTCATCTGAGCCTGTCGACGTGGCCGCTGAGCCAGGAAGAGATC

GCCCGGGCGCTGAAAAAGGCCAAGCCTTCCTATTCTTCTGAGCAAGTGGTCCTGGCTCGT

ATTAACCAGCGACTGTCTGGCTTAAAAGCCGATTTCCGGGTCACCGGTTACACTTCAACC

GACCAGCCGGGCACTCCGCAGGGGTTTGGCCAGACGCAGCCGGCGGATAACTCGTTAGGC

CTGGCATTCAACAACAGTGGCGAGTGGTGGGACGTACACCTTCAGGGCAACGTTGAAGGG

GGAGAACAGATCAGCAACGGATCGCGC

>P35_S805_wzi2_K2

ATGATAAAAATTGCGCGCATTGCCGTGACGTTGGGTTTGCTTTCCTCCCTGGGAGCCCAG

GCTTACGCGGCCGGGTTAGTGGTAAATGACAACGACTTGCGTAACGACCTGGCCTGGCTT

TCCGATCGCGGGGTCATCCATCTGAGCCTGTCGACCTGGCCGCTGAGCCAGGAAGAGATC

TCCCGGGCGCTAAAAAAGGCCAAACCGTCCTATTCTTCTGAACAAGTGGTGCTGGCGCGT

ATTAACCAGCGCCTGTCTGCCTTAAAAGCGGATTTCCGGGTTACCGGCTACACTTCAACC

GATAAGCCGGGCACTCCGCAGGGGTTTGGTCAGACACAGCCGGCAGATAACTCGTTAGGC

CTGGCGTTCAACAACAGCGGCGAGTGGTGGGATGTCCACCTCCAGGGCAACGTCGAAGGA

GGGGAGCGGATCAGCAACGGCTCGCGC

>P35_S806_UK12

ATGATAAAAATTGCGCGCATTGCCGTGACGCTGGGCTTGCTTTCCTCACTGGGAGCCCAG

GCTTACGCGGCCGGGTTAGTAGTAAATGATAACGATCTGCGAAATGACCTTGCCTGGCTT

TCCGATCGCGGGGTCATCCATCTGAGCCTGTCGACCTGGCCGCTGAGCCAGGAAGAGATC

GCCCGGGCGCTAAAAAAAGCTAAACCGTCCTATTCTTCTGAGCAAGTGGTGCTGGCCCGT

ATCAATCAGCGACTGTCTGCTTTAAAAGCCGATTTTCGGGTCACCGGCTATACCTCAACC

GATCAGCCGGGCACTCCGCAGGGGTTTGGTCAGACACAGCCGGCAGATAACTCGTTAGGC

CTGGCGTTTAACAACAGCGGAGAATGGTGGGATGTCCACCTCCAGGGTAACGTCGAAGGG

GGGGAGCGGATCAGCAACGGATCGCGT

>P36_S1436_wzi84_K28

ATGATAAAAATTGCGCGCATTGCCGCGACGTTGGGTTTGCTTTCCTCCCTGGGAGCCCAG

GCTTACGCGGCCGGGTTAGTGGTAAATGACAACGACTTGCGTAACGACCTGGCCTGGCTT

TCCGATCGCGGGGTCATCCATCTGAGCCTGTCGACCTGGCCGCTGAGCCAGGAAGAGATC

GCCCGGGCACTGAAAAAGGCTAAACCGTCCTATTCTTCTGAACAAGTGGTGCTGGCGCGT

ATTAACCAGCGACTGTCTGCCTTAAAAGCGGATTTCCGGGTCACCGGCTACACTTCAACC

GATCAGCCGGGCACTCCGCAGGGGTTTGGCCAGACGCAGCCGGCGGATAACTCGTTAGGC

CTGGCGTTCAACAACAGCGGCGAGTGGTGGGATGTTCACCTGCAGGGCAACATTGAAGGG

GGAGAGCGGATCAGCAACGGATCGCGC

>P36_S1437_wzi84_K28

ATGATAAAAATTGCGCGCATTGCCGCGACGTTGGGTTTGCTTTCCTCCCTGGGAGCCCAG

GCTTACGCGGCCGGGTTAGTGGTAAATGACAACGACTTGCGTAACGACCTGGCCTGGCTT

TCCGATCGCGGGGTCATCCATCTGAGCCTGTCGACCTGGCCGCTGAGCCAGGAAGAGATC

GCCCGGGCACTGAAAAAGGCTAAACCGTCCTATTCTTCTGAACAAGTGGTGCTGGCGCGT

ATTAACCAGCGACTGTCTGCCTTAAAAGCGGATTTCCGGGTCACCGGCTACACTTCAACC

GATCAGCCGGGCACTCCGCAGGGGTTTGGCCAGACGCAGCCGGCGGATAACTCGTTAGGC

CTGGCGTTCAACAACAGCGGCGAGTGGTGGGATGTTCACCTGCAGGGCAACATTGAAGGG

GGAGAGCGGATCAGCAACGGATCGCGC

>P36_S1438_wzi84_K28

ATGATAAAAATTGCGCGCATTGCCGCGACGTTGGGTTTGCTTTCCTCCCTGGGAGCCCAG

GCTTACGCGGCCGGGTTAGTGGTAAATGACAACGACTTGCGTAACGACCTGGCCTGGCTT

TCCGATCGCGGGGTCATCCATCTGAGCCTGTCGACCTGGCCGCTGAGCCAGGAAGAGATC

GCCCGGGCACTGAAAAAGGCTAAACCGTCCTATTCTTCTGAACAAGTGGTGCTGGCGCGT

ATTAACCAGCGACTGTCTGCCTTAAAAGCGGATTTCCGGGTCACCGGCTACACTTCAACC

GATCAGCCGGGCACTCCGCAGGGGTTTGGCCAGACGCAGCCGGCGGATAACTCGTTAGGC

CTGGCGTTCAACAACAGCGGCGAGTGGTGGGATGTTCACCTGCAGGGCAACATTGAAGGG

GGAGAGCGGATCAGCAACGGATCGCGC

>P37_S1032_wzi37_K22.37

ATGATAAAAATTGCGCGCATTGCCGTGACGTTGGGTTTGCTTTCCTCCCTGGGAGCCCAG

GCTTACGCAGCCGGGTTAGTGGTAAATGACAACGACTTGCGTAACGACCTGGCCTGGCTT

TCCGATCGCGGGGTCATCCATCTGAGCCTGTCGACCTGGCCGCTGAGCCAGGAAGAGATC

GCCCGGGCGCTAAAAAAGGCTAAACCGTCCTATTCTTCTGAACAAGTGGTGCTGGCGCGT

ATTAACCAGCGACTGTCTGCCTTAAAAGCGGATTTCCGGGTCACCGGCTACACTTCAACC

GATCAGCCGGGCACTCCGCAGGGGTTTGGCCAGACGCAGCCGGCGGATAACTCGTTAGGC

CTGGCGTTCAACAACAGCGGTGAGTGGTGGGACGTCCACCTTCAGGGCAATGTTGAAGGG

GGAGAGCGGATCAGCAACGGATCGCGC

>P37_S1033_wzi37_K22.37

ATGATAAAAATTGCGCGCATTGCCGTGACGTTGGGTTTGCTTTCCTCCCTGGGAGCCCAG

GCTTACGCAGCCGGGTTAGTGGTAAATGACAACGACTTGCGTAACGACCTGGCCTGGCTT

TCCGATCGCGGGGTCATCCATCTGAGCCTGTCGACCTGGCCGCTGAGCCAGGAAGAGATC

GCCCGGGCGCTAAAAAAGGCTAAACCGTCCTATTCTTCTGAACAAGTGGTGCTGGCGCGT

ATTAACCAGCGACTGTCTGCCTTAAAAGCGGATTTCCGGGTCACCGGCTACACTTCAACC

GATCAGCCGGGCACTCCGCAGGGGTTTGGCCAGACGCAGCCGGCGGATAACTCGTTAGGC

CTGGCGTTCAACAACAGCGGTGAGTGGTGGGACGTCCACCTTCAGGGCAATGTTGAAGGG

GGAGAGCGGATCAGCAACGGATCGCGC

>P37_S1034_wzi37_K22.37

ATGATAAAAATTGCGCGCATTGCCGTGACGTTGGGTTTGCTTTCCTCCCTGGGAGCCCAG

GCTTACGCAGCCGGGTTAGTGGTAAATGACAACGACTTGCGTAACGACCTGGCCTGGCTT

TCCGATCGCGGGGTCATCCATCTGAGCCTGTCGACCTGGCCGCTGAGCCAGGAAGAGATC

GCCCGGGCGCTAAAAAAGGCTAAACCGTCCTATTCTTCTGAACAAGTGGTGCTGGCGCGT

ATTAACCAGCGACTGTCTGCCTTAAAAGCGGATTTCCGGGTCACCGGCTACACTTCAACC

GATCAGCCGGGCACTCCGCAGGGGTTTGGCCAGACGCAGCCGGCGGATAACTCGTTAGGC

CTGGCGTTCAACAACAGCGGTGAGTGGTGGGACGTCCACCTTCAGGGCAATGTTGAAGGG

GGAGAGCGGATCAGCAACGGATCGCGC

>P38_S1187_UK13

ATGATAAAAATTGCGCGCATTGCCGTGACGTTGGGTTTGCTTTCCTCACTGGGAGCCCAG

GCTTACGCGGCCGGGTTAGTGGTAAATGACAACGACTTGCGTAACGACCTGGCCTGGCTT

TCCGATCGCGGGGTCATCCATCTGAGCCTGTCGACGTGGCCGCTGAGCCAGGAAGAGATC

GCCCGGGCGTTAAAAAAGGCCAAGCCGTCTTATTCTTCTGAGCAAGTGGTGCTGGCCCGT

ATCAACCAGCGACTGTCTGCTTTAAAAGCCGATTTCAGGGTCACCGGCTACACCTCAACC

GACCAGCCTGGCACCCCGCAGGGGTTTGGTCAGACGCAGCCGGCGGATAATTCTCTGGGC

CTAGCATTCAACAACAGCGGCGAGTGGTGGGATGTCCACCTGCAGGGCAACGTTGAAGGG

GGAGAACGGATCAGCAACGGATCGCGC

>P38_S1188_UK13

ATGATAAAAATTGCGCGCATTGCCGTGACGTTGGGTTTGCTTTCCTCACTGGGAGCCCAG

GCTTACGCGGCCGGGTTAGTGGTAAATGACAACGACTTGCGTAACGACCTGGCCTGGCTT

TCCGATCGCGGGGTCATCCATCTGAGCCTGTCGACGTGGCCGCTGAGCCAGGAAGAGATC

GCCCGGGCGTTAAAAAAGGCCAAGCCGTCTTATTCTTCTGAGCAAGTGGTGCTGGCCCGT

ATCAACCAGCGACTGTCTGCTTTAAAAGCCGATTTCAGGGTCACCGGCTACACCTCAACC

GACCAGCCTGGCACCCCGCAGGGGTTTGGTCAGACGCAGCCGGCGGATAATTCTCTGGGC

CTAGCATTCAACAACAGCGGCGAGTGGTGGGATGTCCACCTGCAGGGCAACGTTGAAGGG

GGAGAACGGATCAGCAACGGATCGCGC

>P39_S1592_UK14

ATGATAAAAATTGCGCGCATTGCCGTGACGTTGGGTTTGCTTTCCTCCCTGGGAGCCCAG

GCTTACGCGGCCGGGTTAGTGGTAAATGACAACGACTTGCGTAACGACCTGGCCTGGCTT

TCCGATCGCGGGGTCATCCATCTGAGCCTGTCGACGTGGCCGCTGAGCCAGGAAGAGATC

GCCCGGGCGCTGAAAAAGGCCAAACCTTCCTATTCTTCTGAGCAAGTGGTGCTGGCGCGT

ATTAACCAGCGACTGTCTGCCTTAAAAGCGGATTTCCGGGTCACCGGCTACACTTCAACC

GATCAGCCAGGCACTCCGCAGGGGTTTGGTCAGACGCAGTCGGCGGATAATTCGTTAGGC

CTGGCGTACAACAACAGCGGCGAGTGGTGGGATATCCACCTCCAGGGCAACGTCGAAGGA

GGGGAGCGGATCAGCAACGGATCGCGC

>P39_S1593_UK14

ATGATAAAAATTGCGCGCATTGCCGTGACGTTGGGTTTGCTTTCCTCCCTGGGAGCCCAG

GCTTACGCGGCCGGGTTAGTGGTAAATGACAACGACTTGCGTAACGACCTGGCCTGGCTT

TCCGATCGCGGGGTCATCCATCTGAGCCTGTCGACGTGGCCGCTGAGCCAGGAAGAGATC

GCCCGGGCGCTGAAAAAGGCCAAACCTTCCTATTCTTCTGAGCAAGTGGTGCTGGCGCGT

ATTAACCAGCGACTGTCTGCCTTAAAAGCGGATTTCCGGGTCACCGGCTACACTTCAACC

GATCAGCCAGGCACTCCGCAGGGGTTTGGTCAGACGCAGTCGGCGGATAATTCGTTAGGC

CTGGCGTACAACAACAGCGGCGAGTGGTGGGATATCCACCTCCAGGGCAACGTCGAAGGA

GGGGAGCGGATCAGCAACGGATCGCGC

>P39_S1594_UK14

ATGATAAAAATTGCGCGCATTGCCGTGACGTTGGGTTTGCTTTCCTCCCTGGGAGCCCAG

GCTTACGCGGCCGGGTTAGTGGTAAATGACAACGACTTGCGTAACGACCTGGCCTGGCTT

TCCGATCGCGGGGTCATCCATCTGAGCCTGTCGACGTGGCCGCTGAGCCAGGAAGAGATC

GCCCGGGCGCTGAAAAAGGCCAAACCTTCCTATTCTTCTGAGCAAGTGGTGCTGGCGCGT

ATTAACCAGCGACTGTCTGCCTTAAAAGCGGATTTCCGGGTCACCGGCTACACTTCAACC

GATCAGCCAGGCACTCCGCAGGGGTTTGGTCAGACGCAGTCGGCGGATAATTCGTTAGGC

CTGGCGTACAACAACAGCGGCGAGTGGTGGGATATCCACCTCCAGGGCAACGTCGAAGGA

GGGGAGCGGATCAGCAACGGATCGCGC

>P40_S1846_wzi14_K14

ATGATAAAAATTGCGCGCATTGCCGTGACGTTGGGTTTGCTTTCCTCCCTGGGAGCCCAG

GCTTACGCGGCCGGGTTAGTGGTAAATGACAACGACTTGCGTAACGACCTGGCCTGGCTT

TCCGATCGCGGGGTCATCCATCTGAGCCTGTCGACCTGGCCGCTGAGCCAGGAAGAGATC

TCCCGGGCGCTAAAAAAGGCCAAACCGTCCTATTCTTCTGAGCAAGTGGTGCTGGCGCGT

ATTAACCAGCGACTGTCTGCCTTAAAAGCGGATTTCCGGGTCACCGGCTACACTTCAACC

GATCAGCCGGGCACTCCGCAGGGGTTTGGTCAGACACAGCCGGCGGATAACTCGTTAGGC

CTGGCATTCAACAACAGCGGCGAGTGGTGGGATATCCATCTCCAGGGCAACGTTGAAGGG

GGAGAGCGGATCAGCAACGGATCGCGC

>P40_S1847_wzi14_K14

ATGATAAAAATTGCGCGCATTGCCGTGACGTTGGGTTTGCTTTCCTCCCTGGGAGCCCAG

GCTTACGCGGCCGGGTTAGTGGTAAATGACAACGACTTGCGTAACGACCTGGCCTGGCTT

TCCGATCGCGGGGTCATCCATCTGAGCCTGTCGACCTGGCCGCTGAGCCAGGAAGAGATC

TCCCGGGCGCTAAAAAAGGCCAAACCGTCCTATTCTTCTGAGCAAGTGGTGCTGGCGCGT

ATTAACCAGCGACTGTCTGCCTTAAAAGCGGATTTCCGGGTCACCGGCTACACTTCAACC

GATCAGCCGGGCACTCCGCAGGGGTTTGGTCAGACACAGCCGGCGGATAACTCGTTAGGC

CTGGCATTCAACAACAGCGGCGAGTGGTGGGATATCCATCTCCAGGGCAACGTtGAAgGG

GGAGAGCGGATCAGCAACGGATCGCGC

>P40_S1848_wzi14_K14

ATGATAAAAATTGCGCGCATTGCCGTGACGTTGGGTTTGCTTTCCTCCCTGGGAGCCCAG

GCTTACGCGGCCGGGTTAGTGGTAAATGACAACGACTTGCGTAACGACCTGGCCTGGCTT

TCCGATCGCGGGGTCATCCATCTGAGCCTGTCGACCTGGCCGCTGAGCCAGGAAGAGATC

TCCCGGGCGCTAAAAAAGGCCAAACCGTCCTATTCTTCTGAGCAAGTGGTGCTGGCGCGT

ATTAACCAGCGACTGTCTGCCTTAAAAGCGGATTTCCGGGTCACCGGCTACACTTCAACC

GATCAGCCGGGCACTCCGCAGGGGTTTGGTCAGACACAGCCGGCGGATAACTCGTTAGGC

CTGGCATTCAACAACAGCGGCGAGTGGTGGGATATCCATCTCCAGGGCAACGTTGAAGGG

GGAGAGCGGATCAGCAACGGATCGCGC
